# Production and Functional Characterization of Murine Osteoclasts Differentiated from ER-Hoxb8-Immortalized Myeloid Progenitor Cells

**DOI:** 10.1371/journal.pone.0142211

**Published:** 2015-11-03

**Authors:** Frank Zach, Alexandra Mueller, André Gessner

**Affiliations:** Institute of Clinical Microbiology and Hygiene, University Hospital Regensburg, Regensburg, Germany; Faculté de médecine de Nantes, FRANCE

## Abstract

*In vitro* differentiation into functional osteoclasts is routinely achieved by incubation of embryonic stem cells, induced pluripotent stem cells, or primary as well as cryopreserved spleen and bone marrow-derived cells with soluble receptor activator of nuclear factor kappa-B ligand and macrophage colony-stimulating factor. Additionally, osteoclasts can be derived from co-cultures with osteoblasts or by direct administration of soluble receptor activator of nuclear factor kappa-B ligand to RAW 264.7 macrophage lineage cells. However, despite their benefits for osteoclast-associated research, these different methods have several drawbacks with respect to differentiation yields, time and animal consumption, storage life of progenitor cells or the limited potential for genetic manipulation of osteoclast precursors. In the present study, we therefore established a novel protocol for the differentiation of osteoclasts from murine ER-Hoxb8-immortalized myeloid stem cells. We isolated and immortalized bone marrow cells from wild type and genetically manipulated mouse lines, optimized protocols for osteoclast differentiation and compared these cells to osteoclasts derived from conventional sources. *In vitro* generated ER-Hoxb8 osteoclasts displayed typical osteoclast characteristics such as multi-nucleation, tartrate-resistant acid phosphatase staining of supernatants and cells, F-actin ring formation and bone resorption activity. Furthermore, the osteoclast differentiation time course was traced on a gene expression level. Increased expression of osteoclast-specific genes and decreased expression of stem cell marker genes during differentiation of osteoclasts from ER-Hoxb8-immortalized myeloid progenitor cells were detected by gene array and confirmed by semi-quantitative and quantitative RT-PCR approaches. In summary, we established a novel method for the quantitative production of murine bona fide osteoclasts from ER-Hoxb8 stem cells generated from wild type or genetically manipulated mouse lines. These cells represent a standardized and theoretically unlimited source for osteoclast-associated research projects.

## Introduction

Homeostasis and controlled remodeling of bone tissues are maintained by the coupled and balanced action of bone resorbing osteoclasts (OCs) and bone forming osteoblasts [1–3]. The disruption of OC differentiation or activity processes has been described as a key feature in the development of pathological bone abnormalities seen in Paget’s disease of bone (PDB) [4], osteoporosis [5], inflammatory arthritis [6], periodontitis [7], or cancer metastasis to bone [8,9]. For example, patients suffering from PDB display a disturbed OC activity, which is believed to be caused by environmental as well as genetic factors [4]. Thus, familial PDB is associated with mutations in the ubiquitin associated (UBA) domain of sequestosome 1 which encodes p62, a scaffold protein known to be involved in cytokine signaling, and that can serve as a cargo adaptor for polyubiquitinated proteins [4].

OCs are highly differentiated and polarized cells originating from the monocyte-macrophage lineage [10]. Mature OCs can be identified by different biological markers such as tartrate-resistant acid phosphatase (TRAP) staining, multi-nucleation, F-actin ring formation and their unique bone resorbing capacity [10,11]. Besides their primary function in the regulation of bone resorption, OCs are important orchestrators of several other processes, e.g. the regulation of hematopoiesis, bone formation and angiogenesis of blood vessels during bone development [12,13].

In contrast to bone forming osteoblasts, which are mesenchymal-derived cells, OCs are of myeloid origin [13]. *In vitro* differentiation into OCs has successfully been achieved by 1) direct supplementation of primary or cryopreserved spleen and bone marrow (BM)-derived OC precursors, embryonic stem cells (ESCs) or induced pluripotent stem cells (iPSCs) with macrophage colony-stimulating factor (M-CSF) and soluble receptor activator of nuclear factor kappa-B ligand (sRANKL), or 2) by the use of osteoclast-osteoblast co-cultures systems [14–20]. Nevertheless, it is not feasible to cultivate and expand OCs for longer periods of time. In addition, available cell numbers from single differentiation experiments are limited and experimental outcome may be variable. Furthermore, the murine myeloid cell line RAW 264.7, which can also be differentiated into OCs in theoretically unlimited amounts by incubation with sRANKL [21], and which is another standard source of OCs, has to be further manipulated, e.g. by siRNA-treatment in order to perform gene knockdown experiments.

Due to their myeloid origin, OCs might potentially be generated by differentiation of immortalized myeloid progenitors ectopically expressing the homeodomain containing transcription factor *Hoxb8*. Expression of *Hoxb8* (formerly named *Hox-2*.*4*) has long been known to alter growth, differentiation and survival of myeloid cells [22]. Thus, ER-Hoxb8 cells have been developed and characterized as retroviraly transduced murine bone marrow myeloid (BMM) cells, which can be used as conditionally immortalized monocyte-macrophage progenitors [23]. Under the control of β-estradiol, these cells produce Hoxb8 which subsequently inhibits myeloid differentiation and arrests BMMs in an immortalized and self-renewing stem cell (SC) state [23]. In recent years, ER-Hoxb8 SCs have successfully been differentiated into macrophages, neutrophil granulocytes or dendritic cells upon removal of β-estradiol and stem cell factor (SCF) and respective supplementation with M-CSF, granulocyte colony-stimulating factor (G-CSF) or granulocyte macrophage colony-stimulating factor (GM-CSF) [23,24]. Since macrophages and OCs have a common myeloid progenitor and macrophages can been produced in theoretically unlimited amounts using the ER-Hoxb8 SC technique, we intended to investigate the OC differentiation potential of ER-Hoxb8 SCs, which has not been addressed and characterized so far.

The present work establishes a novel protocol for the theoretically unlimited production of mature and functional murine OCs from ER-Hoxb8-immortalized myeloid progenitor cells, and furthermore compares these cells to OCs derived from other conventional sources. Our study describes the differentiation and functional characterization of OCs from immortalized myeloid progenitor cells that were isolated from BM of different wild type (WT) and genetically modified mice. Successfully differentiated OCs show excessive TRAP staining and multi-nucleation as well as functional OC characteristics like F-actin ring formation and resorption activity on dentin discs or a calcium phosphate (CaP) substrate. Thus, immortalized ER-Hoxb8 cells represent a novel and valid source for the potentially unlimited production of functional and biologically active *in vitro* OCs. These cells are a useful tool for enabling and simplifying basic research on OC-associated diseases, as well as for the development of new drugs to manipulate differentiation from OC precursors or functions of mature OCs derived from WT or genetically modified mice.

## Materials and Methods

### Reagents and Media

Recombinant murine M-CSF, sRANKL, GM-CSF, TNF-α, IL-3, IL-4 and IL-15 were purchased from Peprotech Inc. (Rocky Hill, NJ, USA). Other cytokines were obtained from ImmunoTools (Friesoythe, Germany; IL-6) or R&D Systems (Minneapolis, MN, USA; INF-γ). Unless stated otherwise, reagents were bought from Sigma-Aldrich (St. Louis, MO, USA; CaCl_2_, DMSO, β-estradiol, FBS, fibronectin, FITC-phallodin, β-mercaptoethanol, PBS, polybrene, toluidine blue, Triton^™^ X-100), Merck (Darmstadt, Germany; HCl, MgCl_2_, NH_4_Cl) or Roth (Karlsruhe, Germany; AgNO_3_, KHCO_3_, NaCl, Na_2_EDTA, NaHCO_3_, Na_2_HPO_4_, NaOCl). Cell culture media, except for α-MEM (Sigma-Aldrich), were purchased from Life Technologies (Carlsbad, CA, USA). Cell culture supplements penicillin/streptomycin and L-glutamine were obtained from Pan-Biotech (Aidenbach, Germany).

### Animals

Male C57BL/6, and p62/sequestosome 1-deficient C57BL/6 mice [25], BALB/c, and IL-4 receptor- (IL-4R) deficient BALB/c mice [26], and C3H/HeJ mice were kept at the animal facility of the University of Regensburg in a specific pathogen-free environment. Unless stated otherwise, WT mice used for experimental approaches, as well as for propagation of KO mice mentioned above, were obtained from Charles River Laboratories (Wilmington, MA, USA). Mice were sacrificed by CO_2_ asphyxiation at the age of 10–20 wk. Procedures including animals were performed according to the animal guidelines of the animal facility of the University of Regensburg. All animal experiments were approved by the local veterinary authorities and the ethics committee of the District Government of Upper Palatinate (reference 54–2532.1-38/12).

### Isolation of BMCs, generation and cryopreservation of immortalized ER-Hoxb8 SCs

ER-Hoxb8 SCs were generated as described previously [23]. In brief, femurs and tibiae were cut and cells were flushed into petri dishes with RPMI containing 15% FBS, β-mercaptoethanol, penicillin (100 U/ml), streptomycin (100 μg/ml) and L-glutamine (2 mM). Filtered cells (sieve: 100 μm) were centrifuged after a red blood cell lyses step (150 mM NH_4_Cl, 0.1 mM Na_2_EDTA, 10 mM KHCO_3_, pH 7.2–7.4), resolved in medium and plated in cell culture dishes. After incubation for 2 h, non-adherent cells were transferred into a new cell culture dish, supplemented with IL-3 (10 ng/ml), IL-6 (10 ng/ml) and SCF (1:25), and incubated for 2 d until viral transduction. SCF was obtained from CHO-conditioned medium.

Transfection of Phoenix^™^ retroviral packing cell line [27] with both ecotropic packaging and 3HA-ERHBH-HoxB8-Neo (“ER-Hoxb8”) plasmids (provided by St. Jude Children’s Research Hospital, Memphis, TN, USA, and obtained from G. Haecker, Freiburg, Germany) and infection of BM cells with viral supernatants was performed as follows: Phoenix^™^ cells were seeded in complete DMEM at a density of 2 x 10^6^ cells (4.5 x 10^4^ cells per cm^2^) and grown over night in 100 mm dishes. Medium was replaced 30 min prior to CaCl_2_ transfection. Cells were transfected with 10 μg of both plasmids and incubated for 2 d (medium replacement after 16 h). BMMs were then suspended in complete Opti-MEM^®^ containing SCF (1:25) and β-estradiol (2 μM) and seeded at a density of 2 x 10^5^ cells per cm^2^ on fibronectin-coated (0.001% in PBS) 6-well plates. Viral infection of BMMs with Phoenix^™^ supernatants (with polybrene [4 μg/ml]) was achieved by centrifugation for 3 h (1300 x g) at 32°C. Transduced cells were cultured for 5 d and subsequently split every 2–3 d in densities of 2 x 10^4^ cells per cm^2^ into new 6-wells. After three wk of continuous passaging and expansion, cells were harvested and dissolved in culture medium with 20% DMSO for long-term storage in liquid N_2_ (-196°C). Cryopreservation was performed with the computer-controlled freezing system IceCube 15 M from SY-LAB (Neupurkersdorf, Austria).

### In vitro OC and macrophage differentiation of ER-Hoxb8 progenitor cells

ER-Hoxb8 SCs were harvested after 2–3 d in culture (cell density: ~ 1 x 10^6^ cells per 6-well, ~ 1 x 10^5^ cells per cm^2^) by centrifugation (8 min, 300 x g). In order to remove residual SCF and β-estradiol, cells were washed twice with PBS. Cells were resolved in Opti-MEM^®^ (or α-MEM) medium containing either M-CSF (30 ng/ml) alone for macrophage differentiation, or M-CSF (30 ng/ml) together with sRANKL (50 ng/ml) for OC differentiation. For optimal osteoclastogenesis, cells were seeded in 96- and 24-well plates or 100 mm cell culture dishes at densities between 1.6 and 3.2 x 10^4^ cells per cm^2^ and incubated for 5–6 d until the completion of cell fusion and differentiation processes.

### In vitro OC differentiation of primary BMMs and RAW 264.7 macrophages

BMCs were isolated by flushing femurs and tibia from BALB/c mice with complete α-MEM. Filtered cells (sieve: 100 μm) were washed twice with PBS, centrifuged after a red blood cell lysis step (150 mM NH_4_Cl, 0.1 mM Na_2_EDTA, 10 mM KHCO_3_, pH 7.2–7.4), resolved in α-MEM with M-CSF (30 ng/ml) and cultured in cell culture dishes for 1d. Non-adherent cells were used as progenitors for osteoclastogenesis. Cells were resuspended in α-MEM, supplemented with M-CSF (30 ng/ml) plus sRANKL (50 ng/ml) and cultured (1.6 x 10^4^ cells per cm^2^) for 4 d until OC differentiation was completed.

To generate OCs from the macrophage cell line RAW 264.7 (ATCC^®^ TIB-71^™^), cells were cultured in complete DMEM until confluence, then centrifuged, resuspended in complete α-MEM and plated overnight in respective cell culture plates (5.5 x 10^3^ cells per cm^2^). On the next day, cells were supplemented with sRANKL (50 ng/ml) and differentiated for 3–4 days. Culture medium was replaced 48 h after the first addition of sRANKL.

### OC isolation and transfer

For transfer of mature OCs, differentiation was initially performed in tissue culture plates using the respective OC progenitor cell line as described above. Next, culture medium was removed and cells were washed with PBS. Mono-nucleated cells were dissociated with trypsin/EDTA (0.05%/0.02%, Pan-Biotech) for 1 min at 37°C and flushed with PBS. Multi-nucleated cells with higher adherence were detached from cell culture plates by a second addition of trypsin/EDTA (5–10 min). Trypsin was inactivated by the addition of cell culture medium. Cell suspension was centrifuged at 478 x g for 8 min. Cells were suspended with complete medium (M-CSF [30 ng/ml], sRANKL [50 ng/ml]) and re-plated for different OC assays (uncoated or CaP-coated glass coverslips, CaP-coated 24-well plates, dentin discs placed in 24-well plates). Cells on CaP or dentin discs were incubated for a further 48 h prior to characterization or quantification of CaP resorption activity. Isolation of mature ER-Hoxb8-derived macrophages and IL-4-treated OC precursor cells was done in parallel with the isolation of mature OCs as described here.

### TRAP supernatant activity assay and TRAP staining of cells

Detection of TRAP in OCs was performed using the TRAP staining kit from B-Bridge International (Cupertino, CA, USA) according to the manufacturer’s recommendations. For quantification of TRAP activity in cell culture medium samples, 30 μl of each supernatant were mixed with 60 μl of substrate solution and incubated at 37°C. After 3–6 h, optical density was measured at 540 nm with a microplate reader (Bio-Rad Laboratories, Hercules, CA, USA). Absorption values of blank medium samples were subtracted.

### Cytospin^®^ centrifugation and DiffQuik^®^ staining of mature OCs

To examine morphology and cytoplasmic details of OCs derived from different OC progenitor cell lines, mature OCs were isolated as described above and centrifuged (18 x g, 5 min) onto glass microscope slides via a Cytospin^®^ centrifuge. Cells were then air dried overnight and subsequently stained with the DiffQuik^®^ staining kit (Medion Diagnostics International Inc., Miami, FL, USA) according to the manufacturer’s protocol.

### Detection of F-actin ring formation

In order to visualize different podosome and F-actin ring structures in differentiating or mature OCs, untreated or CaP-coated coverslips were used. OCs were washed with PBS and fixed with 4% paraformaldehyde (SERVA, Heidelberg, Germany) for 10 min. Cells were washed with PBS, permeabilized with 0.5% Triton^™^ X-100 for 5 min and washed with PBS. Cells were incubated with FITC-phalloidin (1:1,000) for 30 min. After three washing steps with PBS, coverslips were mounted with SlowFade^®^ Gold Antifade Mountant (Life Technologies). Cells were examined with a BZ-9000 fluorescence microscope (Keyence, Osaka, Japan).

### Pit formation assay with dentin discs

Mature OCs were isolated from 100 mm dishes as described above, resuspended in their respective cell culture medium (with M-CSF [30 ng/ml] and sRANKL [50 ng/ml]), plated on pre-incubated (37°C, 1 h, PBS) devitalized dentin slices (diameter: 5 mm; thickness: 0.3 mm; Immunodiagnostic Systems, Frankfurt am Main, Germany), and placed in 24-well plates. After incubation for 48 h, dentin discs were transferred to new 24-well plates. Discs were washed with PBS and cells were removed by 10 min incubation with 5% aqueous NaClO solution. Cell debris was removed by washing twice with water. Resorption pits were visualized by staining with aqueous 0.1% (w/v) toluidine blue solution for 7 min. Stained dentin discs were washed three times with water, air dried, fixed onto glass microscope slides and examined with a BZ-9000 microscope (Keyence).

### Biomimetic CaP coatings and quantification of CaP resorption

CaP coatings were prepared as described previously [28,29]. The detection and quantification of resorption activity was done with a biomimetic CaP assay [28] using either mature OCs or OCs directly differentiated on CaP-coated plates or glass coverslips. Mature OCs were incubated at 37°C for 48 h, washed twice with PBS and finally removed from plates with NaCl (1 M, in 0.2% Triton^™^ X-100). Culture plates or coverslips were washed twice with water and stained with 5% aqueous AgNO_3_ solution in combination with UV light treatment for 1 h. After three washing steps with water, surface areas were analyzed by bright-field microscopy (BZ-9000, Keyence). Resorption areas were quantified in overview images which were generated by merging 49 (7x7) individual microscopic images (original magnification: 20x) taken from a randomly designated initial point. The overview images covered approximately 10 mm^2^ of the respective 24-well plate. These images were inverted and resulting black dots (threshold size of single area: 100 μm^2^) representing CaP resorption areas were quantified with the BZ-II Analyzer software (Keyence).

### Gene expression profiling

RNA isolation (RNeasy^®^ Micro Kit, Qiagen, Hilden, Germany), RNA quality control and gene array experiments with the Mouse Gene 2.0 ST Array (Affymetrix, Santa Clara, CA, USA) were done at the Kompetenzzentrum für Fluoreszente Bioanalytik (KFB; Regensburg, Germany). Gene expression data were generated at the KFB with Affymetrix *GeneChip Command Console (AGCC)* and *Expression Console* software packages.

### Extraction of RNA, cDNA synthesis, RT-PCR and qRT-PCR

RNA was extracted from ER-Hoxb8-immortalized SCs and ER-Hoxb8-derived OCs with the RNeasy^®^ Mini Kit from Qiagen according to the manufacturer’s recommendations. Reverse transcription with 0.5 μg of isolated RNA was performed using the iScript^™^ Advanced cDNA Synthesis Kit from Bio-Rad Laboratories as stated in the user’s manual.

qRT-PCR analyses were carried out with the LightCycler^®^ 480 SYBR Green I master mix (Roche Diagnostics, Mannheim, Germany) and the ABI PRISM^®^ 7900HT sequence detection system (Life Technologies). The housekeeping gene *Hprt* was used as endogenous control. cDNA segments of indicated genes (Table 1) were amplified from OC or SC cDNAs with a standard PCR protocol and cloned using the pGEM^®^-T easy vector system (Promega, Madison, WI, USA) as stated in the user’s manual. Absolute gene expression was calculated from standard curves of serial dilutions (10^7^–10^2^ copies) of cloned plasmid DNAs. Relative fold changes were normalized to values of respective SCs. The calculation of Ct-values and absolute gene expression data was done with the SDS 2.4 software (Life Technologies). Nucleotide sequences of primers used for semi-quantitative and quantitative RT-PCR depicted in Table 1 were either described previously [30–33] or designed with the Primer3 software. RT-PCR analyses were performed by touchdown PCR with cDNA samples. Plasmid DNAs (20 ng) of respective cloned gene fragments were used as positive control. H_2_O without template DNA served as negative control.

**Table 1:** Primers used for standard, semi-quantitative and quantitative RT-PCR.

**Gene**	**Accession No.**	**Sequence (5’-3’)**	**cDNA, bp**
*Acp5*	NM_007388.3	5’-AAGAACTTGCGACCATTGTTAGC	91
		5’-CGTTCTCGTCCTGAAGATACTGCA	
*Calcr*	NM_007588.2	5’-TCCAACAAGGTGCTTGGGAA	141
		5’-CTTGAACTGCGTCCACTGGC	
*Car2*	NM_009801.4	5’-CTTCGATCCTTGCTCCCTTCTTCCT	150
		5’-GTACGGAAATGAGACATCTGCTCGC	
*Ctsk*	NM_007802.4	5’-ACGTTACTCCAGTCAAGAACCAGGG	139
		5’-GTCACACAGTCCACAAGATTCTGGG	
*Gata1*	NM_008089.2	5’-GGCCCCTTGTGAGGCCAGAGAG	385
		5’-CGCTCCAGCCAGATTCGACCC	
*Gata2*	NM_008090.5	5’-CACTACCTGTGCAATGCCTG	145
		5’-GCCATAAGGTGGTGGTTGTC	
*Hprt*	NM_013556.2	5’-GTTGAATACAGGCCAGACTTTGTTG	163
		5’-GATTCAACTTGCGCTCATCTTAGGC	
*Itgav*	NM_008402.3	5’-TTCGCCGTGGACTTCTTC	81
		5’-CTGGGTCGTGTTCGCTTT	
*Itgb3*	NM_016780.2	5’-CGTCAGCCTTTACCAGAATTATAGTG	109
		5’-TTTCCCGTAAGCATCAACAATG	
*Nfatc1*	NM_001164111.1	5’-TCTGGGAGATGGAAGCAAAGACTGA	82
		5’-TGGTTGCGGAAAGGTGGTATCTCAA	
*Oscar*	NM_175632.3	5’-TGCTGGTAACGGATCAGCTCCCCAGA	309
		5’-CCAAGGAGCCAGAACCTTCGAAACT	

### Statistical analyses

Data are shown as the mean ± SD. Statistical analyses were performed using Student’s *t*-test. Results were considered statistically significant at *p < 0.05, **p < 0.01, and ***p < 0.001.

## Results

### Time course and efficiency of osteoclastogenesis from ER-Hoxb8 SCs

**Fig 1:**
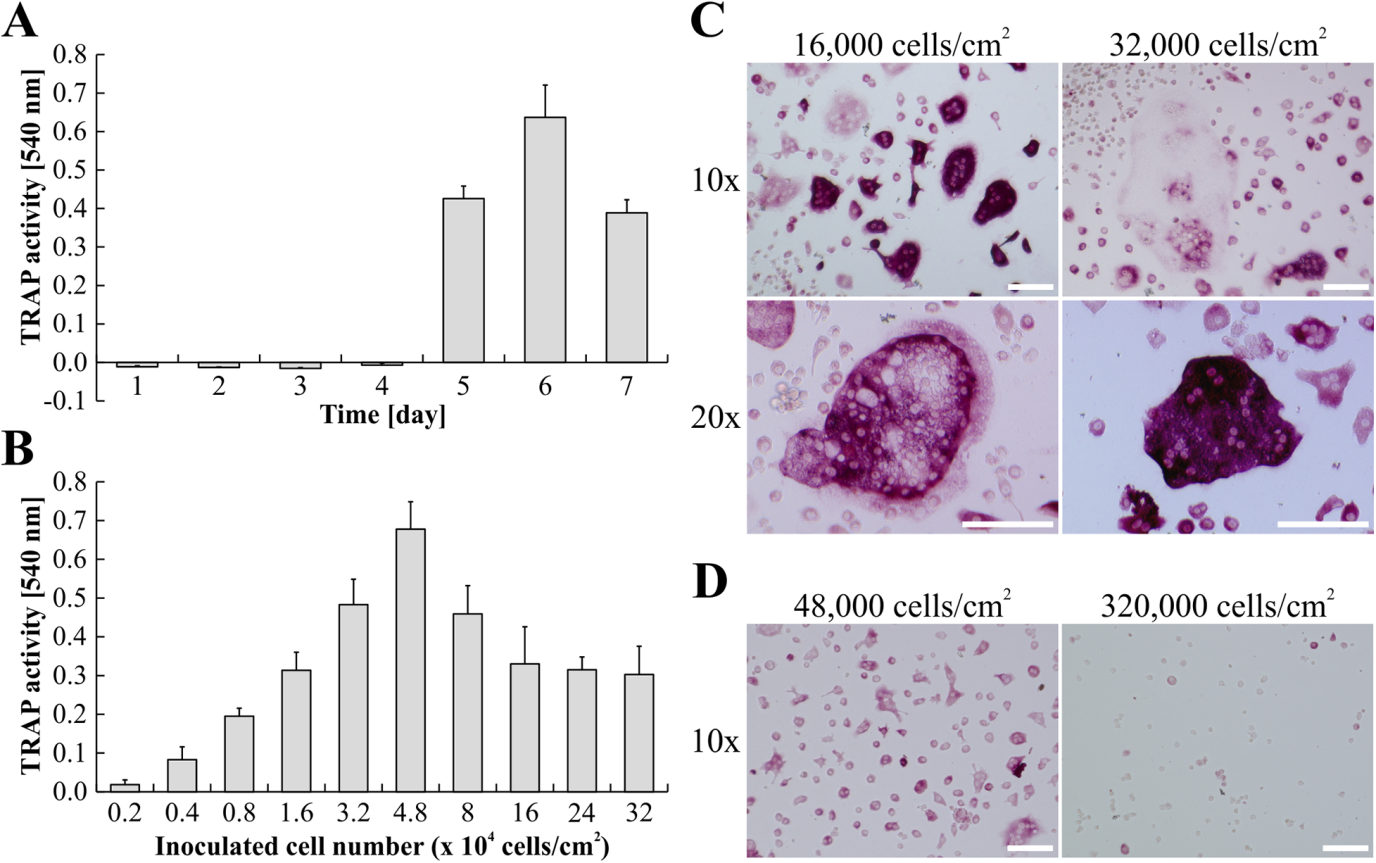
Time course and efficiency of osteoclastogenesis from BALB/c-derived ER-Hoxb8 SCs. (A) The time course of OC differentiation from ER-Hoxb8 SCs (24,000 cells per cm^2^) is illustrated by quantification of TRAP activity in supernatants isolated between d1 and d7. No signs of TRAP activity are detectable in supernatants until d4. Enzyme activity starts to develop at d5 of differentiation and reaches its maximum at d6. Data are shown as the mean ± SD, *n* = 3. (B) SCs were inoculated with indicated cell counts. TRAP activity of supernatants was measured at d5 of M-CSF/sRANKL-induced OC differentiation. Data are displayed as the mean ± SD, *n* = 4. (C, D) Representative bright-field microscopy images of TRAP-stained ER-Hoxb8-derived OCs at d5 of cultivation in the presence of M-CSF and sRANKL. Remarkable differences in cell morphology and multi-nucleation can be seen between wells of intermediary (C) and high cell density (D) inoculations. Scale bars = 100 μm.

As the central aim of this study was to establish OC differentiation from ER-Hoxb8-immortalized myeloid progenitor cells, we first generated ER-Hoxb8 cells from a BALB/c WT mouse as previously described [23]. For OC differentiation, ER-Hoxb8 SCs (24,000 cells per cm^2^) were treated with M-CSF (30 ng/ml) and sRANKL (50 ng/ml). Time course of osteoclastogenesis from these cells was monitored by measuring TRAP activity in culture supernatants between d1 and d7 after cell seeding (Fig 1A). TRAP activity was not detectable in supernatants until d4. Enzyme activity appeared at d5 and reached maximum at differentiation d6 (Fig 1A). OC differentiation for longer time periods, as shown for d7, again resulted in significantly reduced TRAP activity. This indicates a narrow time frame for optimal OC differentiation between d5 and d6 after cell seeding, characterizing ER-Hoxb8-derived OCs, similar to OCs derived from primary BMMs, as relatively short-lived cells.

Since a critical negative correlation between seeded cell number and OC differentiation efficiency has been reported [34], in a next step, progenitor cells were inoculated for OC differentiation in 96-well plates with cell densities ranging from 2,000 up to 320,000 cells per cm^2^. As can be seen in Fig 1B, TRAP activities of cell supernatants at d5 of differentiation showed striking differences depending on initially seeded cell numbers. Highest TRAP activity was observed at intermediary cell densities whereas higher cell densities resulted in a substantial reduction in TRAP activity as an indicator of less efficient OC differentiation (Fig 1B). Fig 1C and 1D show representative examples of OC cell morphology and number of nuclei per cell observed at intermediary cell densities (Fig 1C) compared to high density (Fig 1D) approaches. Thus, by combination of data from TRAP activity assays of supernatants and fixed cells, we were able to depict an optimal seeding density for successful OC differentiation within the range of 16,000–32,000 ER-Hoxb8 SCs per cm^2^.

### Comparison of OC differentiation from ER-Hoxb8 SCs with conventional OC progenitor cell sources

**Fig 2:**
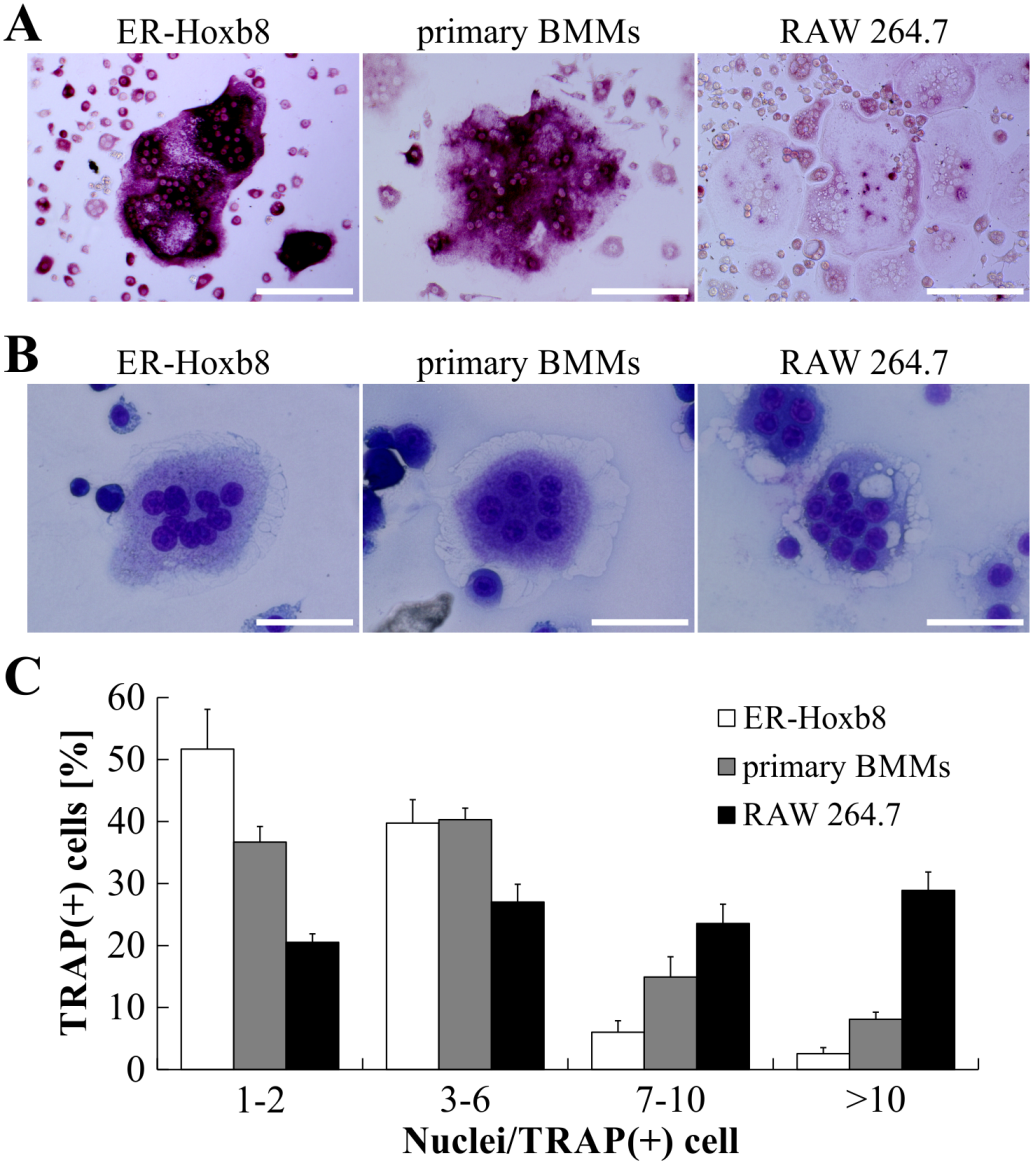
Comparison of OC differentiation from ER-Hoxb8 SCs with conventional OC progenitor sources. (A) Representative microscopy images of OCs derived from ER-Hoxb8 SCs (BALB/c WT), primary BMMs (BALB/c WT) or RAW 264.7 macrophages after formalin fixation and TRAP staining. Scale bars = 150 μm. (B) Examples of bright-field microscopy images of mature OCs after Cytospin^®^ centrifugation and modified Wright-Giemsa staining (DiffQuik^®^). Scale bars = 50 μm. (C) Graphic illustration of percentage of TRAP-positive cells with indicated number of nuclei per cell. OCs derived from RAW 264.7 cells show higher percentage of TRAP-positive cells with 7 or more nuclei. Data are displayed as the mean ± SD, *n* = 4 (4 different 24-wells).

In a next step, we compared osteoclastogenesis from ER-Hoxb8 cells with conventional OC differentiation methods using primary BMMs and the macrophage cell line RAW 264.7 as OC progenitors. TRAP-stained cells in Fig 2A illustrate that no remarkable morphological differences between RAW 264.7-, ER-Hoxb8- and primary BMM-derived OCs were detectable. However, whereas ER-Hoxb8- and primary BMM-derived OCs showed a comparable extent of TRAP staining, intensities of TRAP signals in RAW 264.7-derived OCs were less pronounced, indicating differences between RAW 264.7- and the two BM-derived OC cell cultures.

DiffQuik^®^ staining after Cytospin^®^ centrifugation of mature OCs did not show any striking morphological discrepancies between ER-Hoxb8 generated OCs and conventional OCs (Fig 2B). In order to calculate differentiation efficiencies of the different methods, the number of nuclei per TRAP-positive cells was determined and grouped into several categories (1–2, 3–6, 7–10, >10 nuclei per cell). ER-Hoxb8 cultures displayed a higher percentage of TRAP-positive committed OC progenitors with 1–2 nuclei, whereas freshly isolated BMMs used as OC progenitor resulted in a slightly higher percentage of TRAP-positive cells with 7–10 or >10 nuclei compared to ER-Hoxb8-derived OCs (Fig 2C). Statistical analyses of multi-nucleation in OCs from ER-Hoxb8 and primary BMM progenitors revealed statistically significant differences in categories 1–2, 7–10 and >10, however biological significance is questionable since the percentage of physiological OCs with 3–6 nuclei is indiscriminative. Surface area of OCs derived from different progenitor sources, as exemplified in Fig 2B, were compared but did not exhibit obvious differences in diameter or size of OCs with comparable multi-nucleation status (data not shown). RAW 264.7-derived OCs showed an almost equal distribution of cells in the four different groups and thus a significantly higher percentage of OCs with 7 or more nuclei than both ER-Hoxb8- and BMM-derived OCs.

### Comparison of OC differentiation using ER-Hoxb8 SCs from different mouse strains

**Fig 3:**
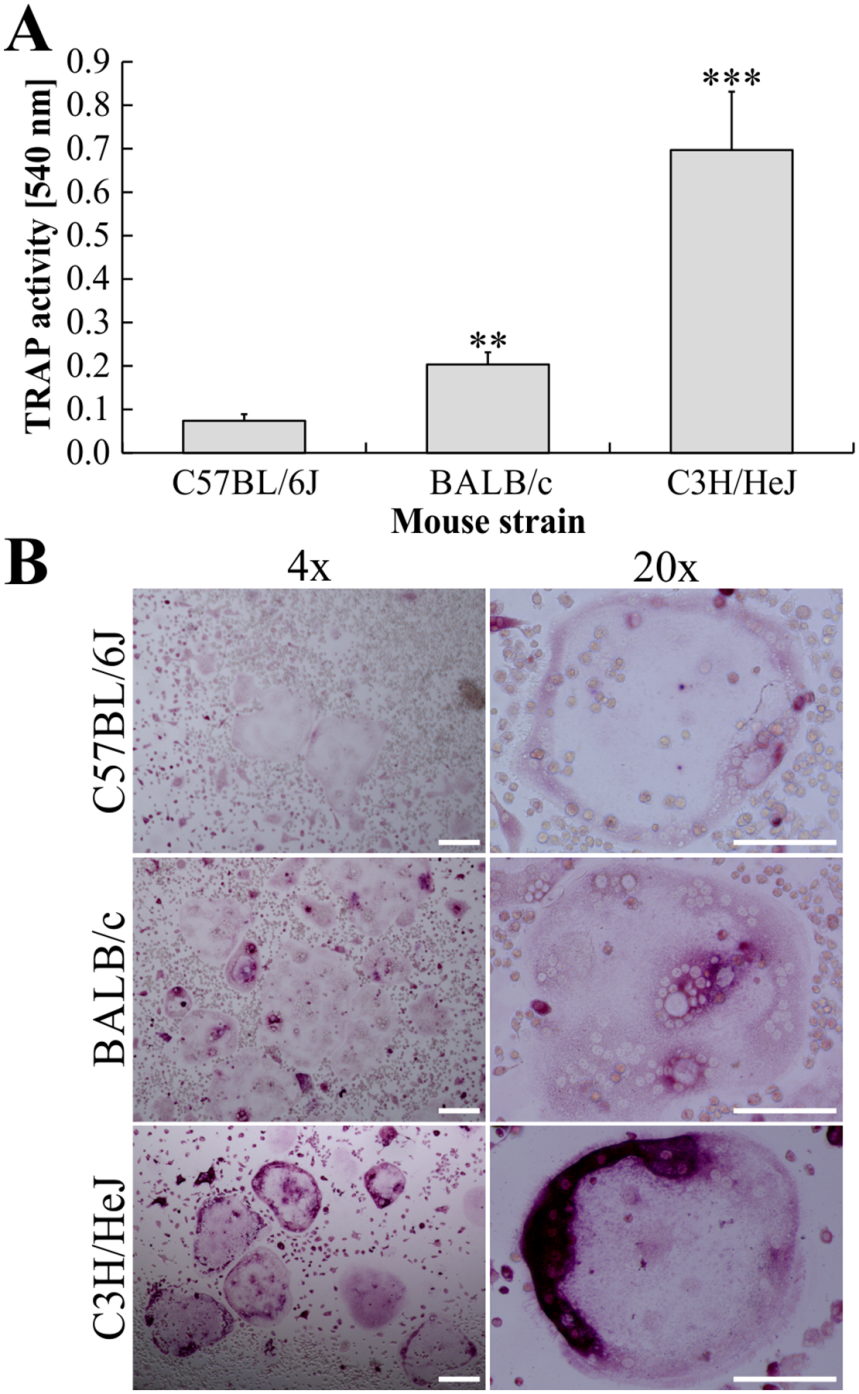
Comparison of osteoclastogenesis using ER-Hoxb8 cells from different WT mouse strains. (A) ER-Hoxb8 OC progenitors (16,000 cells per cm^2^) obtained from BM cells of C57BL/6J, BALB/c and C3H/HeJ WT mice were subjected to osteoclastogenesis with M-CSF and sRANKL. TRAP activity of supernatants was measured at d5 of differentiation. Data are displayed as the mean ± SD, *n* = 3 (three differentiation experiments from identical batches of ER-Hoxb8 SCs). Significant differences in average TRAP activity compared to C57/BL6J-derived OC values are indicated by asterisks (*p < 0.05, **p < 0.01, and ***p < 0.001, Student’s *t*-test). (B) Representative microscopy images of formalin-fixed and TRAP-stained ER-Hoxb8-derived OCs of indicated WT origin. Representative multi-nucleated and TRAP-positive OCs are shown at lower (left column; scale bars = 200 μm) and higher magnification (right column; scale bars = 100 μm).

We next tested whether the osteclastogenesis from ER-Hoxb8 cells is dependent on the genetic background of the SC donor mouse. ER-Hoxb8 cells, generated from the three most widely used WT mouse strains C57BL/6, BALB/c and C3H/HeJ, were each plated at a density of maximal 16,000 cells per cm^2^ (5,000 cells per 96-well; 25,000 cells per 24-well) for OC differentiation. TRAP activity of supernatants was tested at d5 of differentiation. As illustrated in Fig 3A, efficiency of OC differentiation was remarkably different between SCs from analyzed inbred mice. While osteoclastogenesis with C3H/HeJ-derived ER-Hoxb8 cells was more efficient compared to BALB/c cells, supernatants of C57BL/6-derived cells showed comparatively little TRAP activity. These findings were confirmed by microscopic analyses of TRAP-stained cells (Fig 3B). OCs, indicated by TRAP staining and multi-nucleation, were observed in cells of all three mouse strains, however OC yields and intensities of TRAP staining showed differences. Due to these genotype-specific OC differentiation efficiencies, we performed independent ER-Hoxb8 transduction attempts using BM cells from two additional C57BL/6 animals. However, OC differentiation did not show any obvious differences between these three independent C57BL/6-derived cell lines (data not shown) arguing for robust and reproducible mouse strain-specific differences. Thus, in order to use C57BL/6 mouse strains for generation of ER-Hoxb8 stem cells, and to achieve optimal OC differentiation efficiencies for the respective cell line, different parameters such as concentrations of sRANKL and M-CSF, differentiation kinetics and cell densities of OC progenitors will have to be further improved in future differentiation experiments.

### Modulation of OC differentiation and activity using cytokines

**Fig 4:**
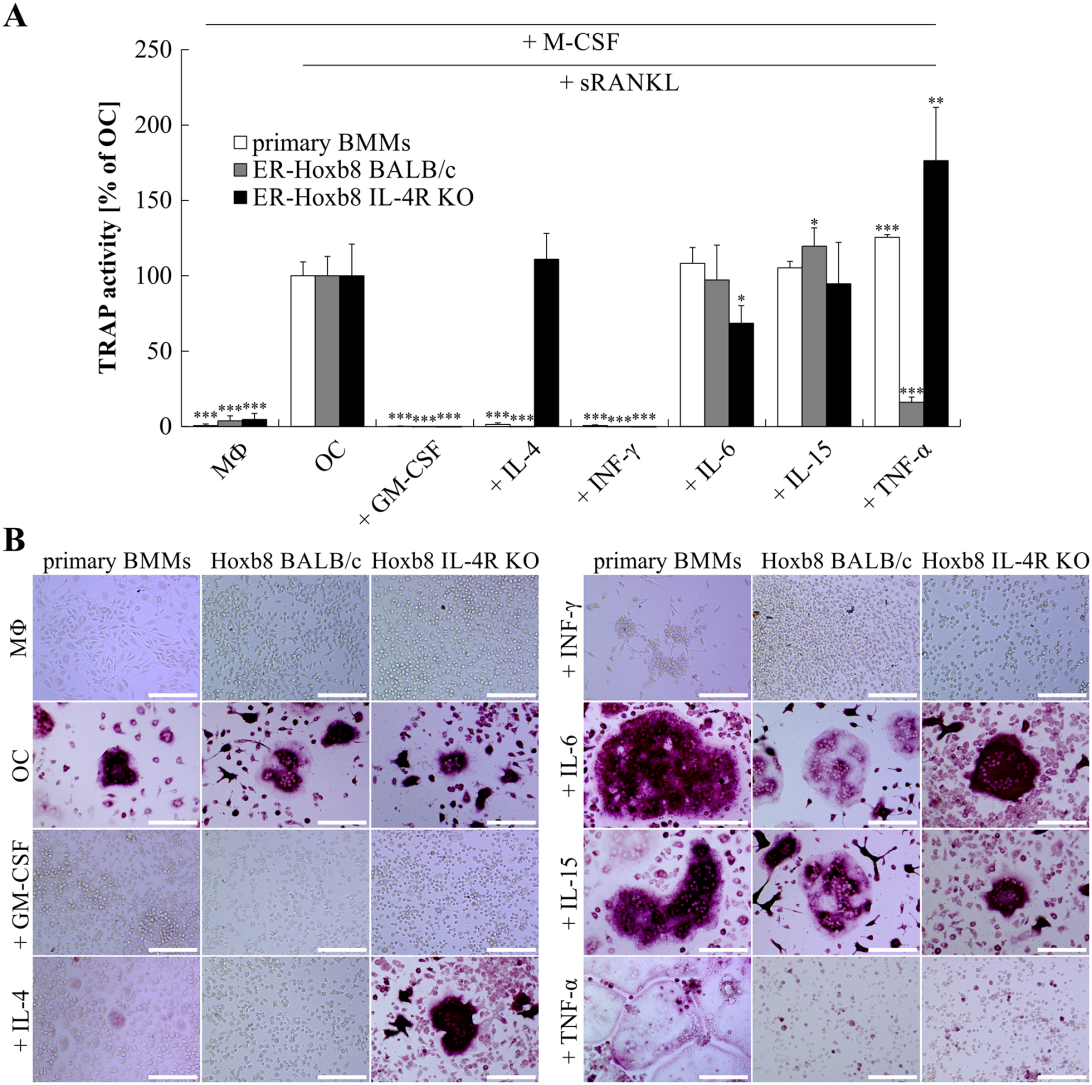
Effect of inhibitory and stimulatory cytokines on OC differentiation of primary BMs or ER-Hoxb8 SCs. (A) BALB/c BMMs or ER-Hoxb8 OC precursor cells of BALB/c and IL-4R KO origin (23,500 cells per cm^2^) were cultured with 1) M-CSF alone (“MФ”), 2) M-CSF and sRANKL (“OC”), or 3) the additional supplementation of indicated cytokines (GM-CSF: 10 ng/ml, remaining cytokines: 20 ng/ml). TRAP activity of supernatants was measured and compared to control differentiation (“OC”). Data are presented as the mean ± SD, *n* = 4. Significant differences of average TRAP activity compared to respective control values (“OC”) are indicated by asterisks (*p < 0.05, **p < 0.01, and ***p < 0.001, Student’s *t*-test). (B) Representative microscopy images illustrating morphology and TRAP staining of formalin-fixed cells after treatment with inhibitory and stimulatory modulators during OC differentiation. M-CSF-treated BMMs and Hoxb8 SCs as well as IL-4-treated BALB/c ER-Hoxb8 and BMMs do not show signs of TRAP staining. IL-4R KO cells are not sensitive to IL-4 and thus show normal OC differentiation potential. Scale bars = 150 μm.

It is well known that OCs, which share similarities with macrophage-like foreign-body giant cells [35], and could be considered to be specialized giant macrophages [12], are sensitive to factors released from immune cells and directly interact with cells of the immune system [12]. To investigate the responsiveness of ER-Hoxb8-derived OCs to different OC-inhibitory cytokines (GM-CSF, IL-4, INF-γ) as wells as OC-stimulatory cytokines (IL-6, IL-15, TNF-α), both were tested during OC differentiation. Fig 4 illustrates TRAP activity of supernatants (Fig 4A) and cells (Fig 4B) derived from either primary BMMs (BALB/c WT) or BALB/c WT and IL-4R KO ER-Hoxb8 SCs at d4 (primary cells) or d5 (ER-Hoxb8 cells) of OC or macrophage (M-CSF) differentiation. As expected, M-CSF-treated cells (“MФ”) showed no detectable TRAP activity (Fig 4A and 4B), while M-CSF in combination with sRANKL (“OC”) resulted in high TRAP activity (Fig 4A and 4B). The addition of the inhibitory cytokine IL-4 (20 ng/ml) completely abolished OC differentiation and therefore TRAP secretion in primary BMM- as well as ER-Hoxb8-derived BALB/c WT cells (Fig 4A and 4B). This is consistent with published findings as, for example, *Yamada* and coworkers were able to show that IL-4 completely blocks osteoclastogenesis of mouse OC precursor cells [36]. Furthermore, we were successful in demonstrating the specificity of this IL-4 effect on ER-Hoxb8 cells since no inhibition of OC differentiation was detectable in IL-4R KO conditions (Fig 4A and 4B). The observed effects of the OC-inhibitory cytokines GM-CSF (10 ng/ml) and INF-γ (20 ng/ml) were comparable in the different cell lines used for OC differentiation (Fig 4A and 4B). TRAP-stained cells in Fig 4B reveal that stimulatory cytokines IL-6 and IL-15 similarly enhanced OC differentiation in all three OC precursor cell lines. However, we noticed that this effect was less pronounced based on the level of TRAP activity in the cell culture supernatants (Fig 4A). TNF-α administration enhanced OC differentiation of freshly isolated BMMs (Fig 4A and 4B), whereas unexpectedly this effect was not visible in TRAP-stained ER-Hoxb8-derived OCs. We further detected a markedly different level of TRAP activity in supernatants of TNF-α-treated IL-4R KO and BALB/c WT ER-Hoxb8-derived OC differentiation conditions. These unexpected differential effects of TNF-α treatment on ER-Hoxb8 cells are currently under in-depth investigation.

### Functional characterization of ER-Hoxb8-derived OCs

**Fig 5:**
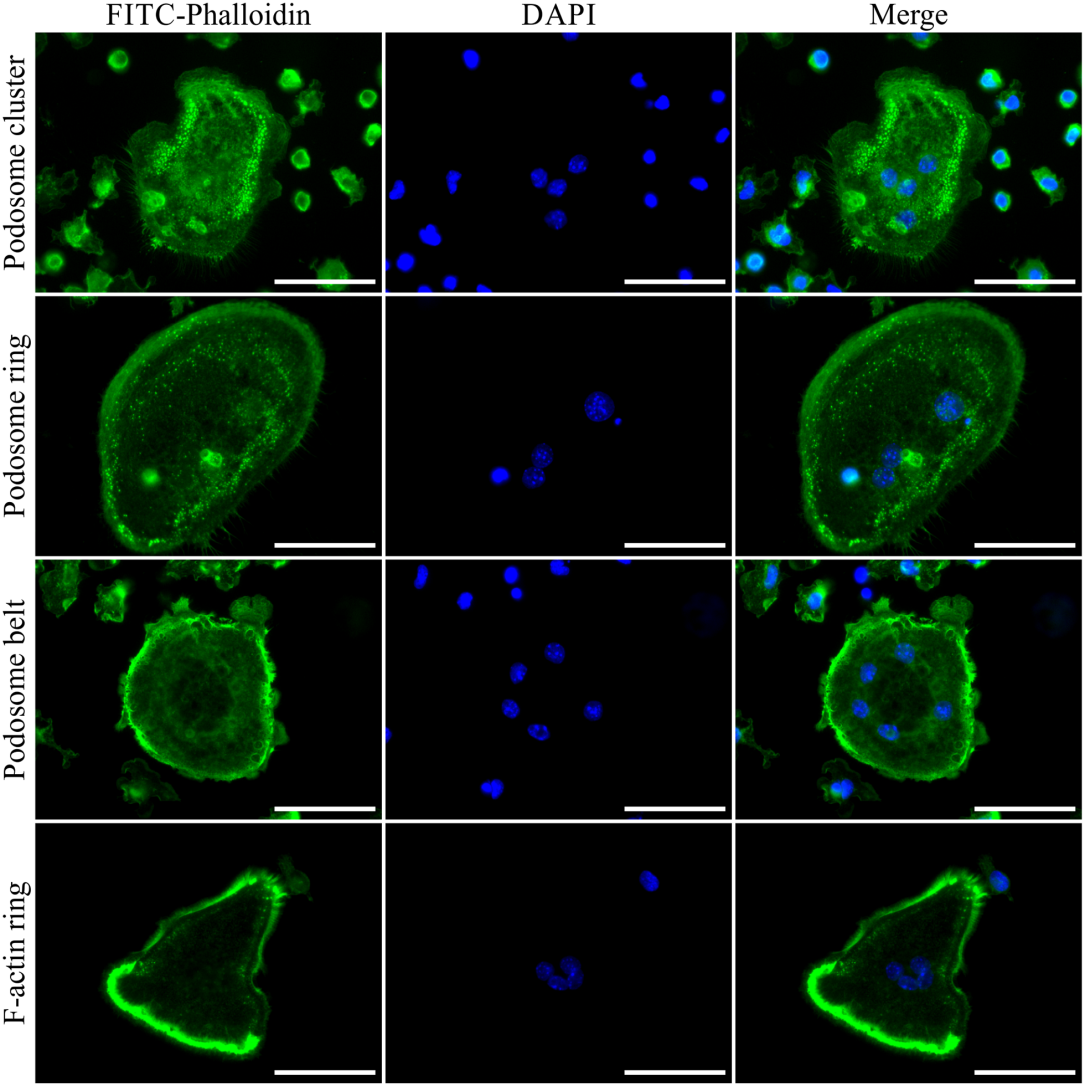
C3H/HeJ ER-Hoxb8-derived OCs show different stages of F-actin ring formation. Visualization of F-actin ring and podosome structures in permeabilized (Triton^™^ X-100) OCs was enabled by interaction of FITC-conjugated phalloidin (green) with F-actin. Nuclei were counterstained with DAPI (blue). Representative fluorescence microscopy images of F-actin structures observed in ER-Hoxb8-derived OCs show examples of podosome cluster, podosome ring and podosome belt in cells differentiated on uncoated glass coverslips. A representative example of fluorescence microscopy images of mature F-actin ring structures in OCs which were differentiated on 100 mm dishes (300,000 cells) and subsequently plated on CaP-coated glass coverslips is shown in the lowest panel. Scale bars = 50 μm.

In order to clarify whether ER-Hoxb8-derived OCs may be designated as a new source of bona fide OCs, we examined the functionality of these cells in comparison to OCs derived from conventional OC progenitor sources. Mature and functional OCs are known to develop an F-actin ring, which is important for the formation of a sealing zone that is associated with OC bone resorption activity [11]. Prior to the arrangement of a mature F-actin ring, actin structures of OCs pass through different intermediate developmental stages called podosome cluster, podosome ring and podosome belt [11,37]. To investigate these structures in ER-Hoxb8-derived OCs, cells were subjected to FITC-phalloidin staining at d5 of OC differentiation. Fig 5 illustrates podosome cluster, podosome ring and podosome belt structures in multi-nucleated cells (C3H/HeJ WT) differentiated on glass coverslips. These structures, which were also visible in BALB/c WT and p62 KO ER-Hoxb8-derived OCs (data now shown), were detectable in comparable quantities and qualities when cells were first differentiated and subsequently plated on biomimetic CaP-coated coverslips (data not shown). It is known that F-actin ring structures, which indicate actively resorbing OCs, are optimally visible on dentin or biomimetic bone substrates, for example on CaP-coated cell culture plates or glass coverslips, and thus fully developed F-actin rings are not likely to be detectable on non-coated plates or coverslips. In order to overcome these limitations, mature OCs, initially differentiated from ER-Hoxb8 progenitors in standard cell culture dishes, were isolated and subsequently plated on CaP-coated glass coverslips. Following incubation for 48 h, cells were stained for F-actin with FITC-phalloidin. Fig 5 (lowest panel) shows a representative multi-nucleated OC containing a heavily stained F-actin ring structure on the cell periphery. Cell movement and signs of OC-associated bone resorption activity at the interface of the F-actin ring are depicted in S1 Fig. The overview of merged bright-field, FICT-phalloidin and DAPI images from ER-Hoxb8-derived OCs (C3H/HeJ WT, p62 KO) points to a directional CaP resorption activity, which is clearly linked to and dependent on its F-actin ring formation on the cell periphery.

**Fig 6:**
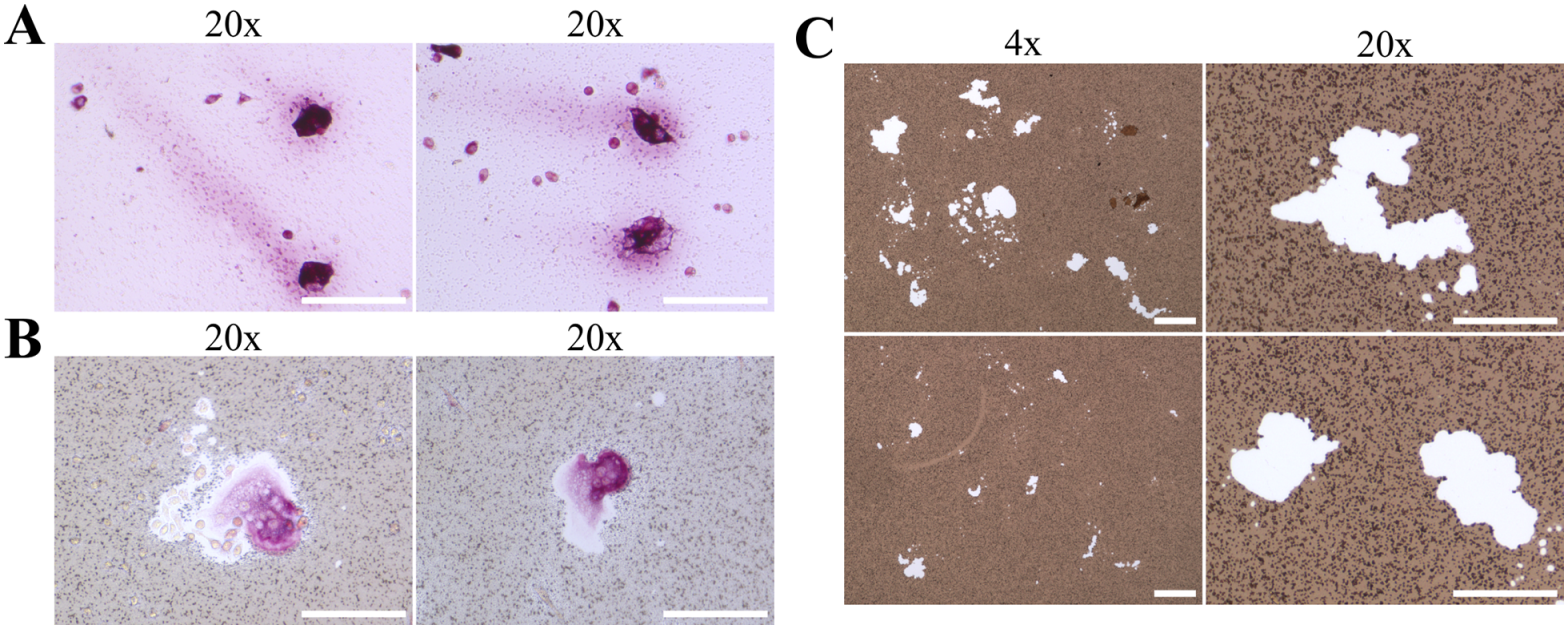
Resorption activity of C3H/HeJ ER-Hoxb8-derived OCs on CaP-coated cell culture plates. (A) Representative bright-field microscopy images of TRAP-stained OCs (seeding density of SCs: 20,000 cells per cm^2^) after 5 d of direct differentiation on CaP-coated plates show intracellular as well as longitudinal tracks of extracellular TRAP enzyme activity. Scale bars = 100 μm. (B) Representative images of TRAP-stained OCs on AgNO_3_-colored CaP-coated cell culture plates surrounded by areas without CaP indicating actively resorbing OCs. OCs were differentiated on uncoated dishes for 5 d, plated on CaP-coated 24-wells and incubated for a further 48 h. Scale bars = 100 μm. (C) Examples of microscopy images at 4x (left column; scale bars = 200 μm) and 20x magnification (right column; scale bars = 100 μm) showing resorption activity of ER-Hoxb8-derived OCs on CaP-coated cell culture plates after AgNO_3_ and UV treatment. Resorption is visualized as white spots lacking CaP and remaining unstained despite application of AgNO_3_.

For further functional characterization of ER-Hoxb8-derived OCs, we analyzed bone resorption, another hallmark of mature OCs. As a starting point, ER-Hoxb8 OC progenitors were directly differentiated in biomimetic CaP-coated cell culture plates for 5 d as previously shown. Subsequently, adherent cells on CaP substrate were stained to determine their TRAP activity (Fig 6A), or alternatively removed in order to visualize remaining CaP deposits via AgNO_3_ treatment. Although OCs showed substantial extra- and intracellular TRAP deposits (Fig 6A), almost no white spots, as an indicator of active resorption, were visible after AgNO_3_ treatment of CaP-coated cell culture plates (data not shown). Thus, we next tried to improve resorption activity of ER-Hoxb8-derived OCs by performing a first differentiation step of ER-Hoxb8 OC precursors in cell culture dishes, followed by the subsequent re-plating of mature OCs in CaP-coated cell culture plates. Fig 6B shows representative TRAP-positive multi-nucleated OCs surrounded by areas clear of CaP coating, as an indicator of resorbing cells. In parallel, we stained for CaP deposits after previous removal of OCs from culture plates and detected multiple white holes of varying size on the CaP coatings (Fig 6C) indicative of resorption activity of multi-nucleated OCs. Interestingly, substantial resorption activity on CaP-coated plates was also detected with OCs from ER-Hoxb8 SCs generated from p62-deficient mice (S2 Fig), facilitating, for example, the investigation of the influence of p62-deficiency on the development and progress of the OC-associated PDB phenotypes.

**Fig 7:**
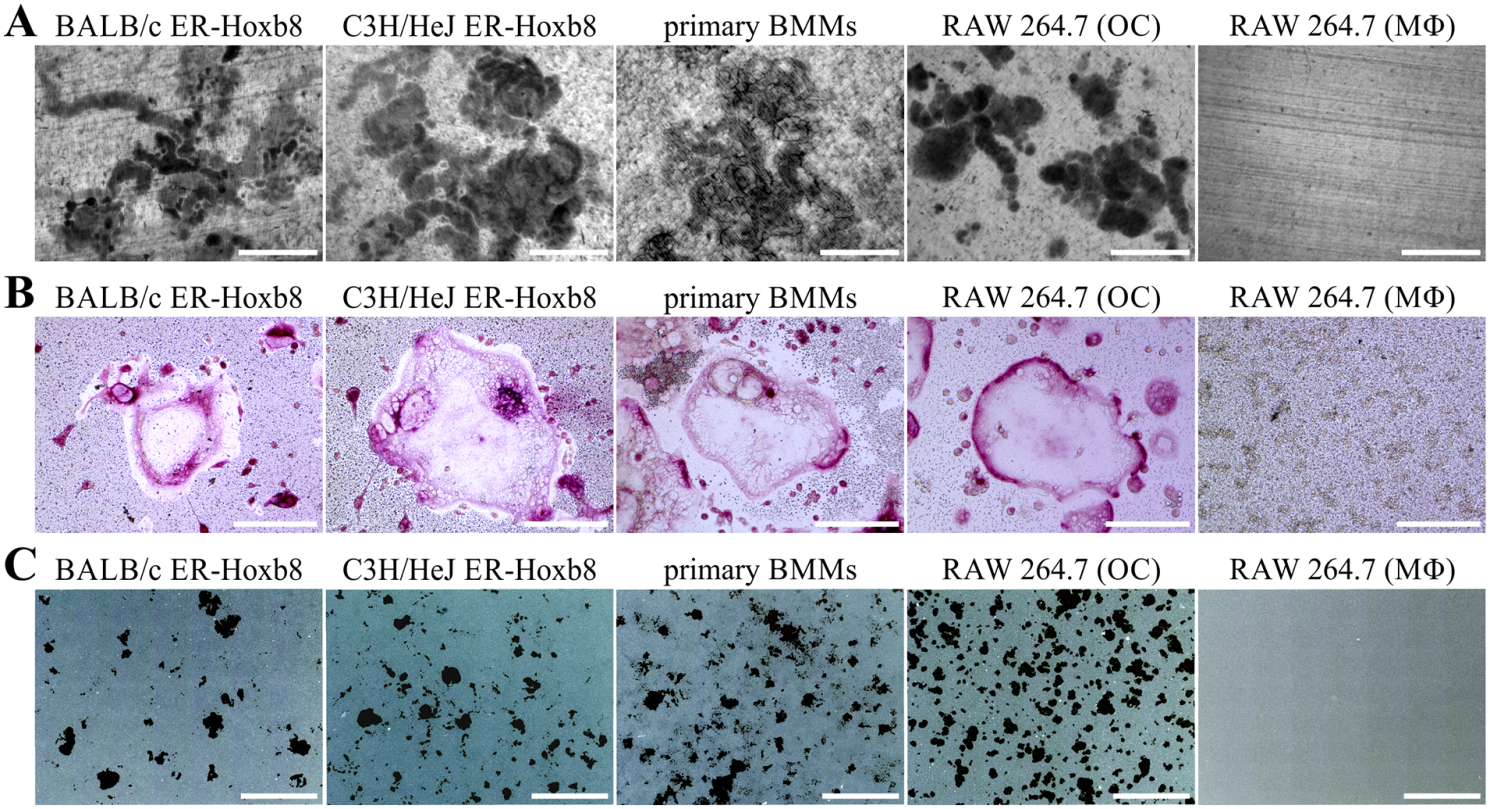
Resorption activity of ER-Hoxb8-derived OCs compared to conventional OCs. (A) Representative microscopy images of pit formation assays performed with dentin discs and mature OCs from indicated sources. Resorption pits were visualized after removal of cells and toluidine blue staining. RAW 264.7 macrophages (“MΦ”) were used as negative control. Scale bars = 400 μm. (B) Microscopy images of mature OCs re-plated in CaP-coated cell culture plates after formalin-fixation and TRAP plus AgNO_3_ staining. Scale bars = 100 μm. (C) Representative examples of merged and inverted overviews of 24-well cell culture plates obtained from 7x7 individual microscopic images at 20x magnification. Resorption areas are visible as black spots. Scale bars = 1 mm.

We next compared the resorption activity of ER-Hoxb8-derived OCs with OCs differentiated from primary BMM or RAW 264.7 cells. Resorption pits on toluidine blue-stained dentin discs (Fig 7A), TRAP-stained cells on Ag-treated CaP substrate surfaces (Fig 7B), as well as CaP-associated (black dots) resorption activities (Fig 7C) of different OCs indicated no obvious qualitative differences between the OC differentiation methods that were analyzed in this study. Furthermore, the inverted overview pictures from 24-well cell culture plates (black dots in Fig 7C) were quantified by Keyence BZ-II software in 4 independent wells of 24-well cell culture plates. The average CaP surface resorbed by OCs of ER-Hoxb8 C3H/HeJ origin (11.0 x 10^5^ μm^2^) was comparable to OCs derived from primary BMMs (12.5 x 10^5^ μm^2^), whereas ER-Hoxb8 BALB/c-derived OCs showed a slightly lower resorption activity (5.5 x 10^5^ μm^2^). OCs differentiated from RAW 264.7 macrophages displayed the highest CaP resorption values of all cell lines that were analyzed in our experiments (33.5 x 10^5^ μm^2^). In summary, CaP resorption of ER-Hoxb8-derived OCs is comparable to conventional OC sources and previously published results [28].

In order to rule out nonspecific matrix loss or CaP resorption, e.g. as a consequence of acidification of the culture medium, several differentiation experiments with ER-Hoxb8 myeloid progenitor cells were performed as resorption controls. After removal of SCF and estradiol, ER-Hoxb8 cells were treated 1) with M-CSF (macrophage differentiation), 2) with M-CSF and sRANKL in combination (OC differentiation) or 3) with M-CSF, sRANKL and IL-4 (20 ng/ml, inhibition of normal OC differentiation). Differentiated and adherent cells were isolated at d6 and seeded on CaP-coated cell culture plates for a further 48 h. Medium alone (data not shown) and ER-Hoxb8 SCs with no known bone resorption activity were used as negative controls. Multi-nucleated and TRAP-positive cells with active resorption were present only in wells with OC differentiation conditions (S3 Fig). Furthermore, the development of resorption pits characterized by the absence of brown CaP deposits after AgNO_3_ staining was completely inhibited by IL-4 (S3 Fig).

Collectively, our results show that multi-nucleated and TRAP-positive ER-Hoxb8-derived cells exhibit typical characteristics of functional bona fide OCs, e.g. F-actin ring formation and bone resorption activity on dentin discs and CaP-coated cell culture plates.

### Analysis of gene expression profiles during OC differentiation

**Fig 8:**
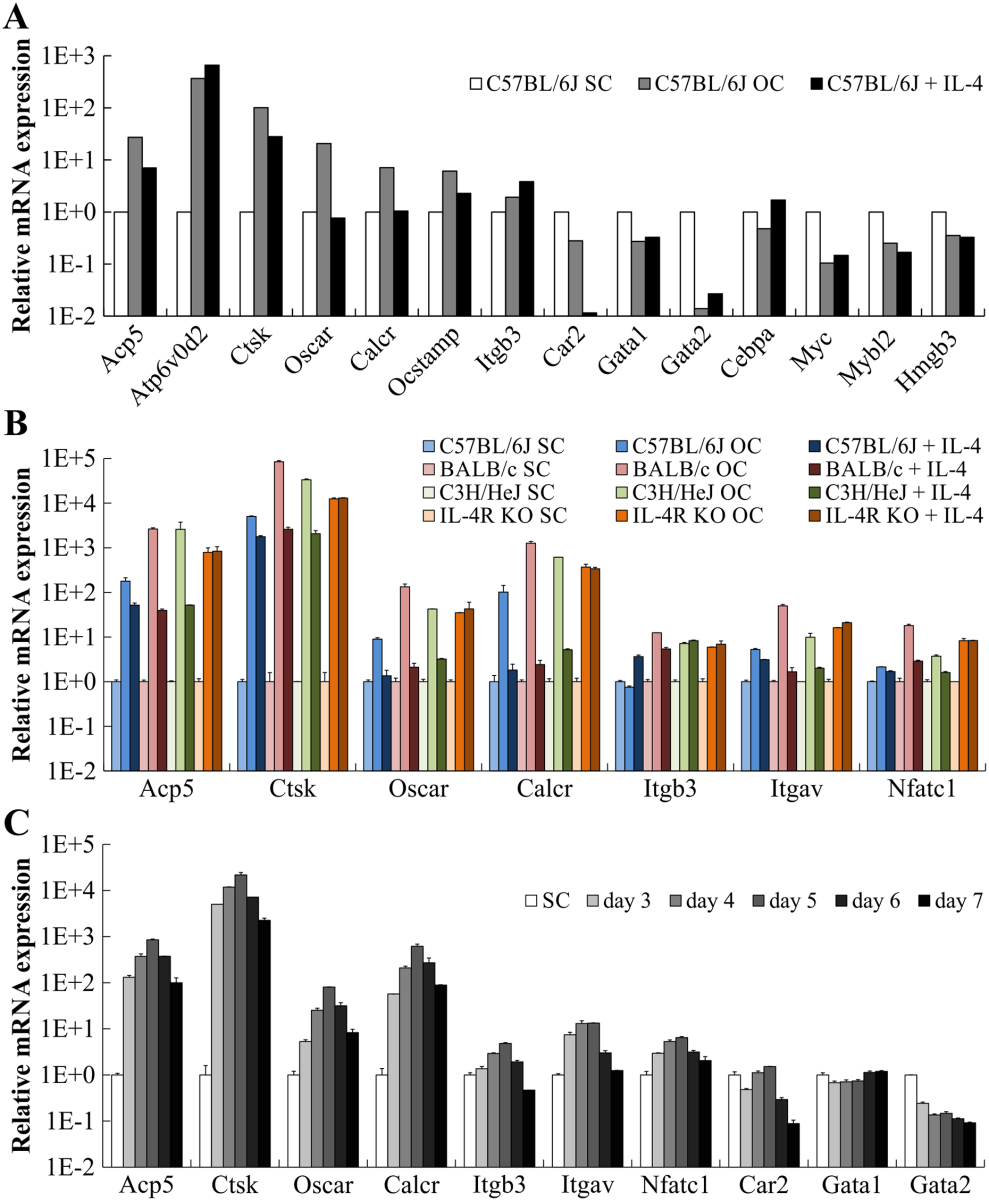
Gene expression profiles of ER-Hoxb8-derived OCs and ER-Hoxb8 SCs. (A) Relative mRNA expression of selected OC or SC marker genes probed with cDNA originating from C57BL/6J ER-Hoxb8 SCs compared to cDNAs of ER-Hoxb8-derived OCs and IL-4-treated OC differentiations. Expression was determined by Mouse Gene 2.0 ST Array from Affimetrix. (B) qRT-PCR analyses of indicated OC and SC marker genes illustrate OC differentiation. RNAs were isolated from ER-Hoxb8 cells of indicated WT or IL-4R KO origin after 5 d under OC differentiation conditions (20,000 cells per cm^2^). Gene expression of respective SC cultures served as reference control. Data are mean ± SD of technical duplicates. Each sample was pooled together from three 24-wells. Housekeeping gene *Hprt* was used for normalization of samples. (C) qRT-PCR analyses of indicated OC and SC marker genes were performed with samples isolated from BALB/c cells after 3–7 d under OC differentiation conditions. Gene expression of respective SC culture served as reference control. Data are mean ± SD of technical duplicates. Samples were pooled together from three 24-wells. Expression of *Hprt* was used for normalization. Note log scale on y axis (A-C).

We next aimed to analyze OC differentiation of ER-Hoxb8 myeloid progenitor cells at the level of gene expression. Since p62 mice, which will be used in our future experiments to generate and further characterize PDB-related ER-Hoxb8-derived OCs, are on a C57BL/6 background [25], we decided to initiate gene expression experiments with C57BL/6-derived cells. Thus, expression profiles from p62 (data not shown) and C57BL/6 ER-Hoxb8 SCs as well as from the respective ER-Hoxb8-derived OCs were compared by applying Mouse Gene 2.0 ST Array from Affimetrix. Relative mRNA expression levels of a selection of OC (*Acp5* [TRAP], *Atp6v0d2*, *Ctsk*, *Oscar*, *Calcr*, *Ocstamp*, *Itgb3*, *Car2*) and SC marker genes (*Gata1*, *Gata2*, *Cebpa*, *Myc*, *Mybl2*, *Hmgb3*) in cells of C57BL/6 WT origin are summarized in Fig 8A, respectively. In OCs, we detected a strong upregulation of almost all analyzed OC markers up to 366-fold (*Atp6v0d2*) compared to untreated ER-Hoxb8 myeloid progenitors (Fig 8A, white and gray bars). Absolute gene expression level (data not shown) of *Car2*, encoding the OC-associated carbonic anhydrase 2, was already high in ER-Hoxb8 SCs and not further elevated in OCs. However, administration of OC-inhibitory cytokine IL-4 completely abolished *Car2* expression pointing to its importance for OC functionality (Fig 8A). IL-4 also reduced (*Acp5*, *Ctsk*, *Ocstamp*) or abolished (*Oscar*, *Calcr*) expression of most other analyzed OC-specific genes, which is in line with the strong inhibitory effect of IL-4 on OC differentiation (Fig 8A, black bars). In contrast, IL-4 further enhanced *Atp6v0d2 and Itgb3* expression compared to ER-Hoxb8 SCs or ER-Hoxb8-derived OCs (Fig 8A). Besides the observed upregulation of OC-specific genes, OCs showed substantial downregulation of SC marker genes, especially *Gata2* and *Myc* (Fig 8A), which further indicated the genetic reprogramming during OC differentiation. Of interest, however, was that these effects on SC marker genes, except for *Cebpa*, were not reversed by IL4 treatment (Fig 8A).

In order to validate the gene array data presented in Fig 8A, we subsequently used SYBR^®^ Green based qRT-PCR with primer pairs covering several SC- and OC-specific genes. Relative expression levels of OC markers *Acp5*, *Ctsk*, *Oscar*, *Calcr*, *Itgb3*, *Itgav*, *Nfatc1* and *Car2*, as well as SC markers *Gata1* and *Gata2* (Fig 8B and S4 Fig) were analyzed in C57BL/6, BALB/c and C3H/HeJ WT plus IL-4R KO ER-Hoxb8-derived OCs and compared to the respective ER-Hoxb8 SC line. Expression levels of OC marker genes *Acp5*, *Ctsk*, *Oscar*, *Calcr*, *Itgb3*, *Itgav* and *Nfatc1* were extensively increased during OC differentiation in all cell lines (Fig 8B). While IL-4 treatment of WT cells, except for *Itgb3*, significantly reduced the increased gene expression of OC marker genes to an extent between OC and SC values, IL-4 had, as expected, no inhibitory effect on OC differentiation in IL-4R KO-derived OCs (Fig 8B). Expression of SC marker *Gata2* strongly decreased during OC differentiation and the addition of IL-4 caused only marginal effects on *Gata2* expression (S4 Fig). Additional experiments were performed to analyze the time course of gene expression during OC differentiation from d3 to d7 using BALB/c WT ER-Hoxb8 cells (Fig 8C). Expression of OC marker genes *Acp5*, *Ctsk*, *Oscar*, *Calcr*, *Itgb3*, *Itgav* and *Nfatc1* strongly increased during OC differentiation and reached a maximum level at d5 (Fig 8C). In contrast, *Car2* and *Gata2* gene expression levels continuously decreased during OC differentiation and *Gata1*-mRNA levels varied only marginally (Fig 8C).

**Fig 9:**
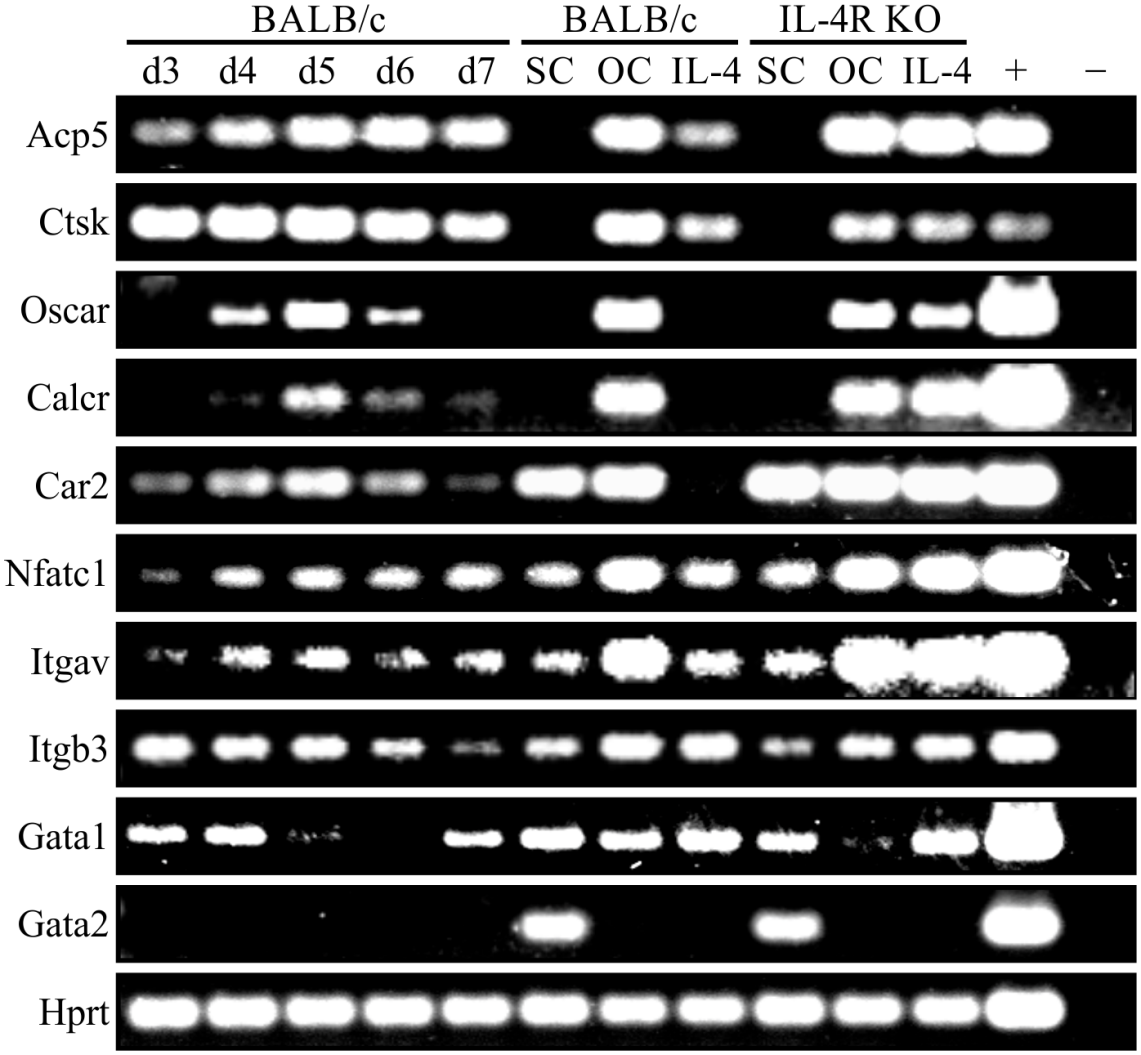
RT-PCR analyses of gene expression in ER-Hoxb8-derived OCs and ER-Hoxb8 SCs. Agarose gels of RT-PCR analyses showing expression of selected OC and SC marker genes in BALB/c- or IL-4R KO-derived SCs, OCs or IL-4-treated OC differentiation. Time course of gene expression between d3 and d7 of OC differentiation is illustrated in BALB/c-derived cells. Gene expression of OC marker genes is maximal at d5 of differentiation and decreases again at d6 and d7. Uniform *Hprt* expression is shown as housekeeping control. Plasmid DNAs served as positive controls (+); water was used as negative control (-).

Semi-quantitative RT-PCR analyses using a selection of samples from the qRT-PCR mentioned above, further confirmed the qRT-PCR results (Fig 9). We observed a strong increase in OC marker gene expression, with maximum signals at d5, and a decrease in ER-Hoxb8 SC-specific gene expression during osteoclastogenesis. Furthermore, the inhibitory effect of IL-4 on BALB/c WT-, but not on IL-4R KO-derived OC progenitors, can be estimated by comparing the intensities of the respective DNA bands after completed PCR reactions. In summary, by using quantitative as well as semi-quantitative RT-PCR analyses, we were able to prove the OC differentiation potential of ER-Hoxb8 SCs on the level of gene expression.

## Discussion

Previous OC-associated research focused preferentially on osteoclast-osteoblast co-culture systems or the *in vitro* differentiation of freshly isolated spleen and BM-derived OC precursors [14, 15, 16, 18, 19, 20]. Alternatively, murine RAW 264.7 cells, which can be differentiated into OCs by simple incubation with sRANKL, have been used in basic approaches [21]. Further progress has also been made recently as protocols became available for the cryopreservation of OC precursors [15], for the isolation and cultivation of OCs from mouse BM [38], and for the OC differentiation from ESC and iPSC sources [17]. However, despite their benefits for basic OC research, these methods have numerous drawbacks. Direct *ex vivo* isolation of OCs is limited by very low yields and cells cannot be cultivated for longer time periods. Repeated experimental attempts with freshly isolated progenitor cells and their resulting differentiated OCs are both highly time and animal consuming, with subsequent findings suffering from a relatively high variability. Cryopreservation of untreated OC precursors from mouse BM and spleen cells results in a limited half-life [15]. OC differentiation from RAW 264.7 OC progenitors is theoretically possible in unlimited amounts, but it is also artificial and cells have to be genetically manipulated for targeted gene knockdown [39]. Different RAW 264.7 cell clones may furthermore have extremely variable OC differentiation potential [40,41]. In addition, experiments with RAW 264.7-derived OCs may be influenced by the inherent genetic background (BAB/14), or the transforming molecular mechanisms operative in this Abelson leukemia virus-transformed mouse monocyte-macrophage cell line.

In the present study, we therefore tested the *in vitro* OC differentiation potential of ER-Hoxb8-immortalized murine myeloid progenitor cells in comparison to primary BMM preparations as well as RAW 264.7 cell cultures in order to establish a new source of unlimited and inexpensive *in vitro* generated OCs. We have proven that OC differentiation is possible with ER-Hoxb8 cells isolated from different WT and genetically manipulated mouse lines. These multi-nucleated ER-Hoxb8-derived cells show OC characteristics such as TRAP staining of supernatants and cells, F-actin ring formation and bone resorption activity on dentin slices as well as on CaP-coated cell culture plates. Furthermore, we were able to show a significantly increased expression of OC- and a decreased expression of SC-specific genes during differentiation of OCs from ER-Hoxb8 cells. Therefore, our results expand the findings of *Wang* and *Redecke* in which the authors were able to differentiate functional macrophages, neutrophil granulocytes and dendritic cells from ER-Hoxb8-immortalized SCs [23,24]. Thus, ER-Hoxb8 progenitor cells display a broad myeloid differentiation potential for different cell types of the respective lineages, including OCs, that are difficult to isolate or cultivate directly in high numbers *ex vivo* and without pre-activating them by using a conventional isolation procedure.

In the present study, differentiation efficiencies and OC yields using ER-Hoxb8 SCs were slightly lower but still comparable to both primary BMM preparations and RAW 264.7 cells. However, osteoclastogenesis was found to depend on the genetic background of the SC donor mice. Since LPS is known to inhibit osteoclastogenesis [42], our finding that C3H/HeJ-derived cells, with natural mutation in the TLR-4 receptor [43], showed the highest OC differentiation capacity, which may be due to a lack of TLR-4-mediated signals. Our future experiments will test this hypothesis.

When functionality of the *in vitro* generated OCs was tested, bone resorption capacity and cytokine responsiveness, among other functions, were found to display typical OC features. However, some cytokine effects were only moderate and not as pronounced as expected or as previously described. This moderate impact on OCs could be due to indirect or artificial effects of cytokines, which may not be fully visible in our *in vitro* system composed of only OCs and no other contaminating cell types. For example, IL-6 is known to inhibit OCs by direct treatment, but stimulate them in cooperation with osteoblasts [44]. Moreover, some cytokines may only be effective at supra-physiological concentrations. Beyond this, we detected a significantly different TRAP activity level in supernatants of TNF-α-treated IL-4R KO and BALB/c WT ER-Hoxb8-derived OCs compared to conventional OCs differentiated from primary BMMs. These highly reproducible and very interesting observations concerning the effects of TNF-α on OC precursor cells under these specific OC differentiation conditions were defined as a starting point for further ongoing experiments with IL-4R KO and BALB/c WT ER-Hoxb8-derived OCs.

The present study describes a reproducible and simple method for the quantitative generation of murine *in vitro* OCs, which, since they share important characteristics, may substitute for the use of repeated batches of fresh BM preparations, thus reducing the time, cost and number of animals required. On the basis of our results, *in vitro* generated ER-Hoxb8-derived OCs are available in theoretically unlimited amounts. Due to this simple availability, these cells can be used for different applications, e.g. large drug screenings to detect substances which influence the differentiation and resorption activity of OCs. Since genetic reprograming of ER-Hoxb8 SCs is already completed at d5 of OC differentiation (Figs 8 and 9), we assume that even approaches targeting the effects of estrogens on mature ER-Hoxb8-derived OCs may be feasible. However, since we currently cannot completely rule out any non-physiological effects of estrogens on mature ER-Hoxb8 OCs, this point will have to be addressed in future investigations for final clarification.

As a further advantage of ER-Hoxb8-derived OCs, signaling events induced by various pharmaceuticals or mediators, e.g. cytokines, may be analyzed more easily by phosphoprotein western blot or proteome analyses requiring relatively high amounts of cellular protein. In addition, the investigation of the effects on OC biology caused by mutations or gene deficiencies may be improved by the use of ER-Hoxb8-derived OCs. For example, our results clearly show the feasibility of this new approach for examining the OC differentiation and CaP resorption capacity of p62-deficient ER-Hoxb8-derived OCs, whereas previously only an incomplete siRNA-mediated knockdown of p62 in RAW 264.7 cells yielded the opposite results [39]. Thus, *in vivo* effects, e.g. mutations or deletions in p62, which are associated with PDB [4], and which are known to ultimately enhance OC resorption activity, may be detected more readily with the ER-Hoxb8-dependent OC differentiation model presented here.

Nonetheless, some limitations of the ER-Hoxb8 technique should also be mentioned. Since statistically significant effects between different mouse lines can be detected most accurately by the use of larger cohorts in *in vivo* experiments, ER-Hoxb8-derived OCs should not replace but initiate and support *in vivo* experiments. In this context, the use of ER-Hoxb8-derived OCs may be a good starting point for initial experiments with variable test parameters and conditions. Promising *in vitro* results may then be applied to larger *in vivo* experiments in order to replicate and expand upon previously established data from ER-Hoxb8-derived OCs.

Since the delivery of ER-Hoxb8 expression constructs into murine BM cells is accomplished by the use of the retroviral transduction procedure described above, this technique implicates the potential danger of a random inactivation of genes in the host genome. However, for several reasons we believe that this legitimate concern can be rebutted. First of all, we used several independent cell lines which so far did not show any sign of inactivation of specific genes. Furthermore, we observed high retroviral transduction efficiencies of cells by the parallel use of a GFP-expression construct (data not shown). This leads us to the assumption that ER-Hoxb8 cell lines are not the result of a single clonal event, but can be described as a diverse mixture of independent transduction incidents. As a consequence of these observations, transduction events resulting in a severe impairment of single cells may be overcompensated by a vast majority of viable and functionally unimpaired cells.

In summary, we established a novel and feasible method for differentiating OCs from ER-Hoxb8-immortalized myeloid progenitor cells. These OCs were further characterized on a functional and genetic level. We compared ER-Hoxb8-derived OCs with conventionally produced *in vitro* OCs and showed that they share typical standard OC characteristics. To the best of our knowledge, this is the first study using ER-Hoxb8 cells for OC differentiation and we propose that such *in vitro* generated ER-Hoxb8-derived murine OCs represent a new significant and quantitative source of bona fide OCs and a valid tool for future OC-associated research.

## Conclusions

The present work establishes a novel *in vitro* method for the quantitative generation of functional murine OCs from ER-Hoxb8-immortalized myeloid progenitor cells. These new bona fide OCs offer an inexpensive and valid alternative to conventional OCs differentiated from primary BMC preparations, which are both time and animal consuming, or from RAW 264.7 cell lines, which are artificial and may have variable OC differentiation efficiencies. Thus, ER-Hoxb8-derived OCs have the potential to improve and facilitate future OC-associated research projects.

## Supporting Information

S1 Fig Resorption activity of ER-Hoxb8-derived OCs from different mouse lines. Images of merged bright-field, FICT-phalloidin and DAPI channels show the resorption activity of ER-Hoxb8-derived OCs from C3H/HeJ (upper panel) or p62 KO mice (lower panel) on CaP-coated cell culture plates. Areas lacking CaP as a consequence of OC resorption are schematically visualized by black borders in microscopy images taken in bright-field channel mode (first column). Direction of resorption and motility of OCs can be estimated from FITC-phalloidin-stained F-actin ring structures (green) concentrated on the cell periphery. Nuclei were counterstained with DAPI (blue). Scale bars = 50 μm.
(TIF)

S2 Fig Resorption activity of ER-Hoxb8-derived OCs of p62 KO origin. (A) Representative images of large (left) and small (right) multi-nucleated TRAP-stained p62 KO OCs on AgNO_3_-colored CaP-coated cell culture plates. Scale bars = 100 μm. (B) Examples of microscopic images at lower (left column; scale bars = 200 μm) and higher magnification (right column; scale bars = 100 μm) showing resorption of p62 KO ER-Hoxb8-derived OCs on CaP-coated cell culture plates after removal of cells, addition of AgNO_3_, and treatment with UV light. (C) Representative examples of merged and inverted overviews of 24-well cell culture plates obtained from 7x7 individual microscopic images at 20x magnification. Resorption areas are visible as black spots. Scale bars = 1 mm.
(TIF)

S3 Fig Resorption activity of ER-Hoxb8 SCs, ER-Hoxb8 macrophages, ER-Hoxb8 OCs and IL-4-treated OC differentiations. Mature cells that had subsequently been cultured on CaP substrate for 48 h are visualized by TRAP staining in combination with AgNO_3_ and UV treatment (upper panel). After removal of cells and AgNO_3_ staining, resorption areas show up as white spots (lower panel). CaP resorption is exclusively present in ER-Hoxb8-derived OCs. Scale bars = 100 μm.
(TIF)

S4 Fig Gene expression of *Car2*, *Gata1* and *Gata2* in ER-Hoxb8-derived OCs and ER-Hoxb8 SCs. qRT-PCR data of genes *Car2*, *Gata1* and *Gata2*. RNAs were isolated from ER-Hoxb8 cells of indicated WT or IL-4R KO origin after 5 d under OC differentiation conditions (20,000 cells per cm^2^). Gene expression of respective SC cultures served as reference control. Data are mean ± SD of technical duplicates. Each sample was pooled together from three 24-wells. Housekeeping gene *Hprt* was used for normalization of samples.
(TIF)
