# Supplementary figures and images for: Production and Functional Characterization of Murine Osteoclasts Differentiated from ER-Hoxb8-Immortalized Myeloid Progenitor Cells
Source: PLoS One. 2015 Nov 3;10(11):e0142211. doi: 10.1371/journal.pone.0142211 (PMC4631598; doi:10.1371/journal.pone.0142211)

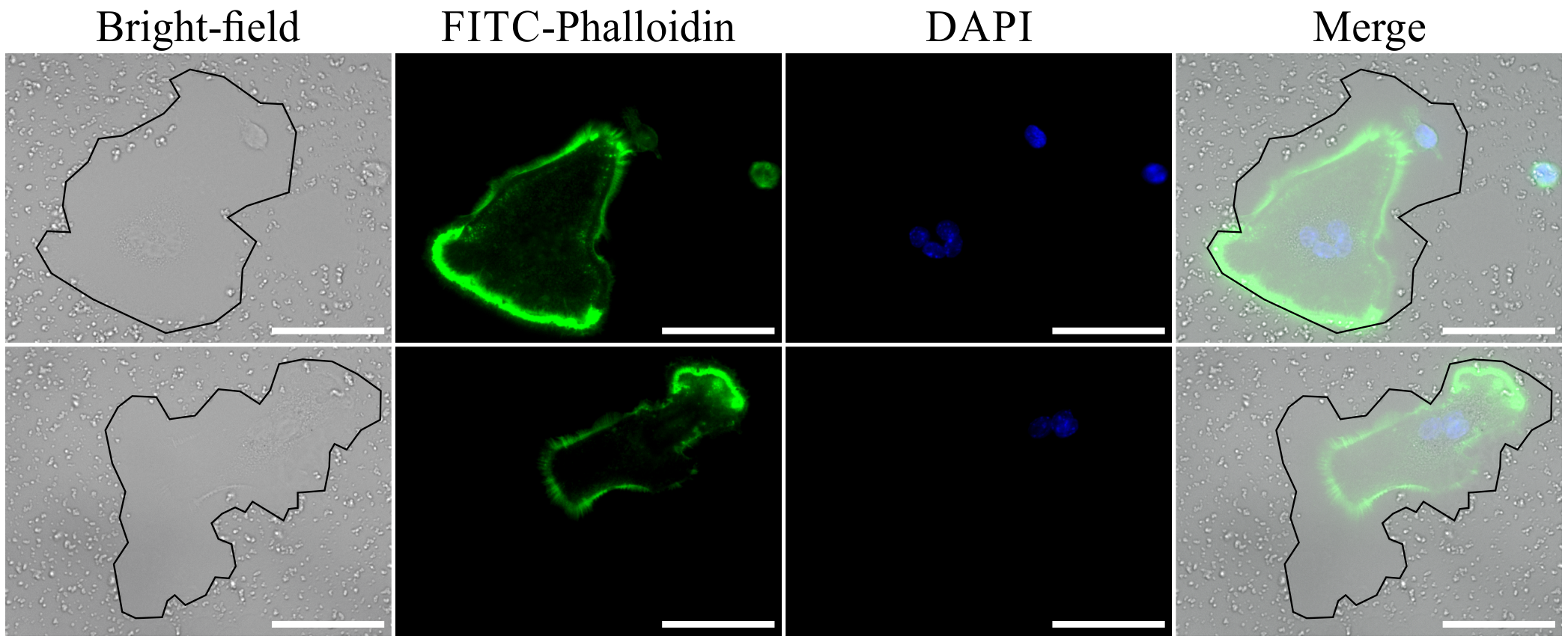

Supplement: S1 Fig — Images of merged bright-field, FICT-phalloidin and DAPI channels show the resorption activity of ER-Hoxb8-derived OCs from C3H/HeJ (upper panel) or p62 KO mice (lower panel) on CaP-coated cell culture plates. Areas lacking CaP as a consequence of OC resorption are schematically visualized by black borders in microscopy images taken in bright-field channel mode (first column). Direction of resorption and motility of OCs can be estimated from FITC-phalloidin-stained F-actin ring structures (green) concentrated on the cell periphery. Nuclei were counterstained with DAPI (blue). Scale bars = 50 μm. (TIF) [file pone.0142211.s001.tif]

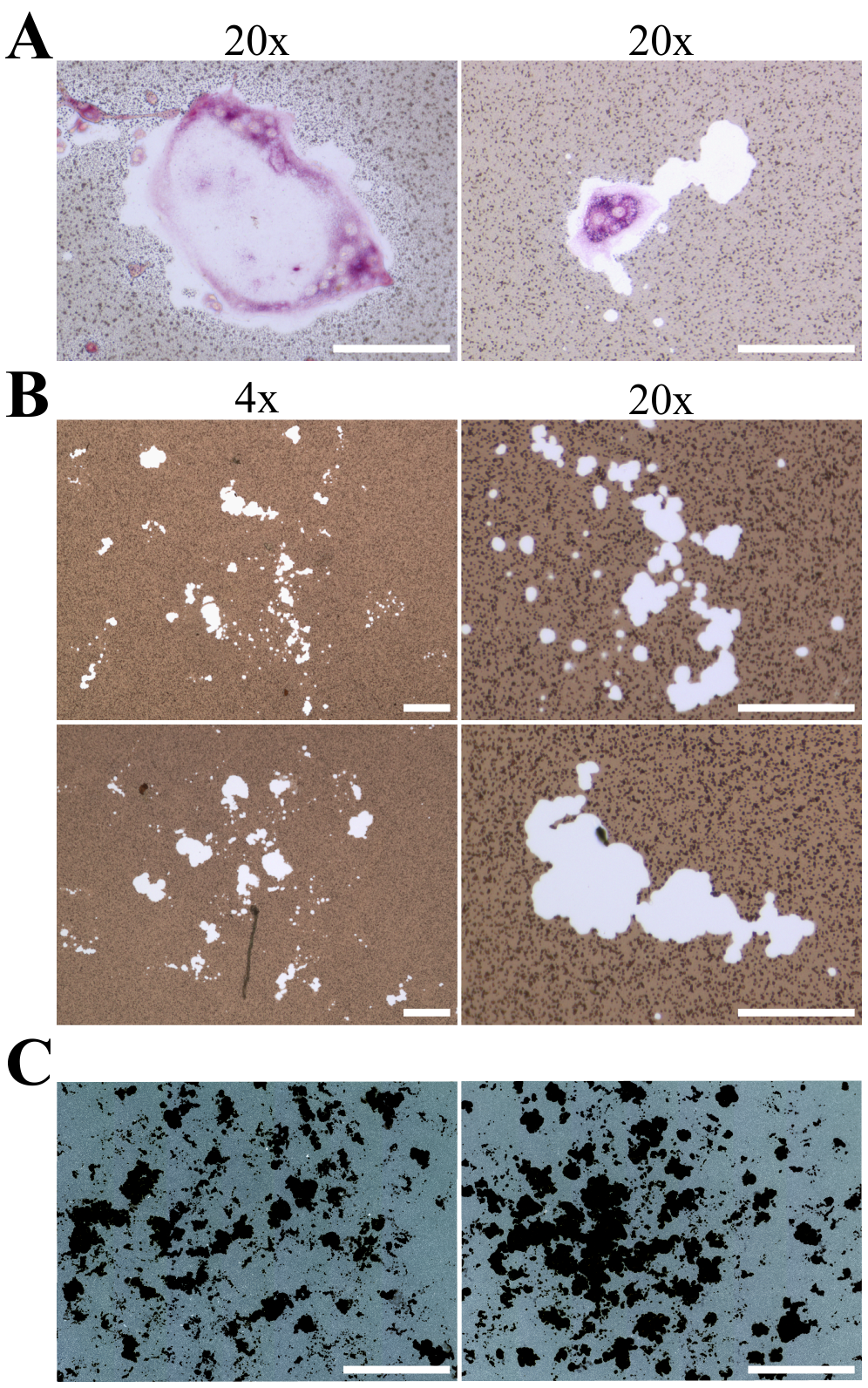

Supplement: S2 Fig — (A) Representative images of large (left) and small (right) multi-nucleated TRAP-stained p62 KO OCs on AgNO3-colored CaP-coated cell culture plates. Scale bars = 100 μm. (B) Examples of microscopic images at lower (left column; scale bars = 200 μm) and higher magnification (right column; scale bars = 100 μm) showing resorption of p62 KO ER-Hoxb8-derived OCs on CaP-coated cell culture plates after removal of cells, addition of AgNO3, and treatment with UV light. (C) Representative examples of merged and inverted overviews of 24-well cell culture plates obtained from 7x7 individual microscopic images at 20x magnification. Resorption areas are visible as black spots. Scale bars = 1 mm. (TIF) [file pone.0142211.s002.tif]

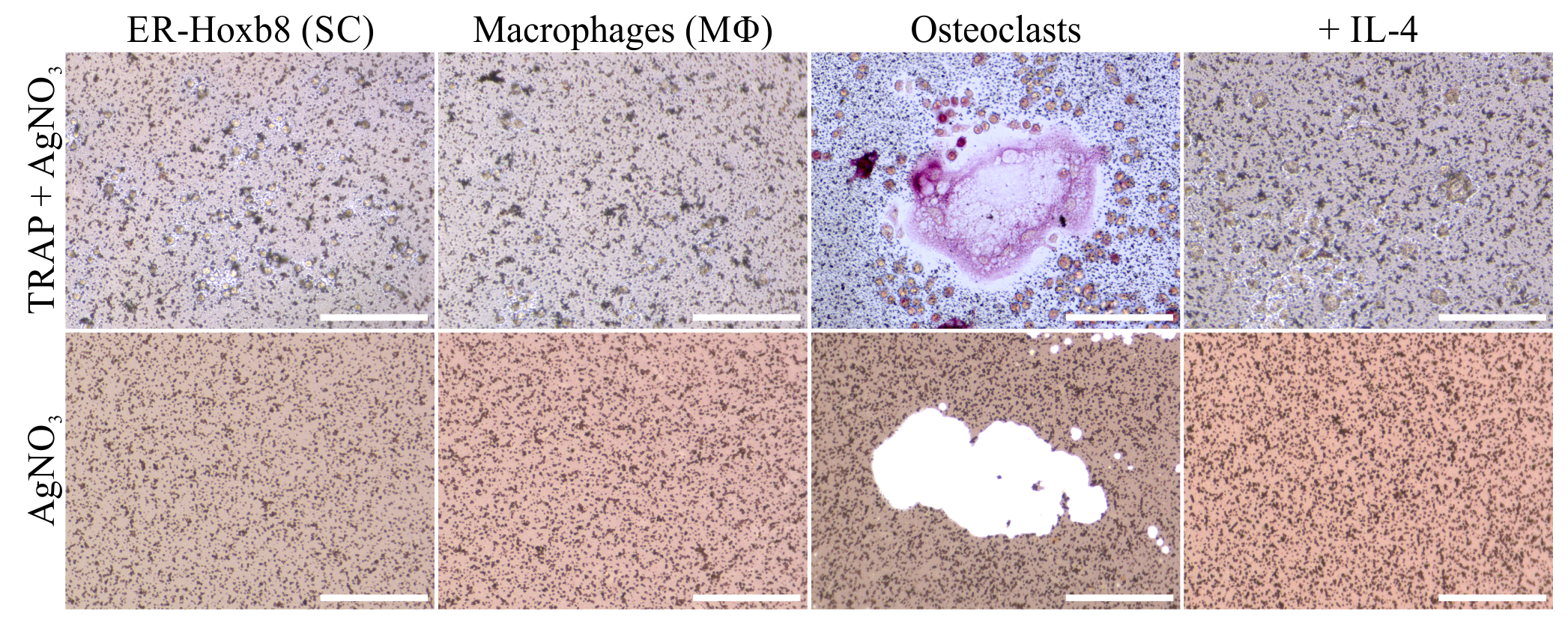

Supplement: S3 Fig — Mature cells that had subsequently been cultured on CaP substrate for 48 h are visualized by TRAP staining in combination with AgNO3 and UV treatment (upper panel). After removal of cells and AgNO3 staining, resorption areas show up as white spots (lower panel). CaP resorption is exclusively present in ER-Hoxb8-derived OCs. Scale bars = 100 μm. (TIF) [file pone.0142211.s003.tif]

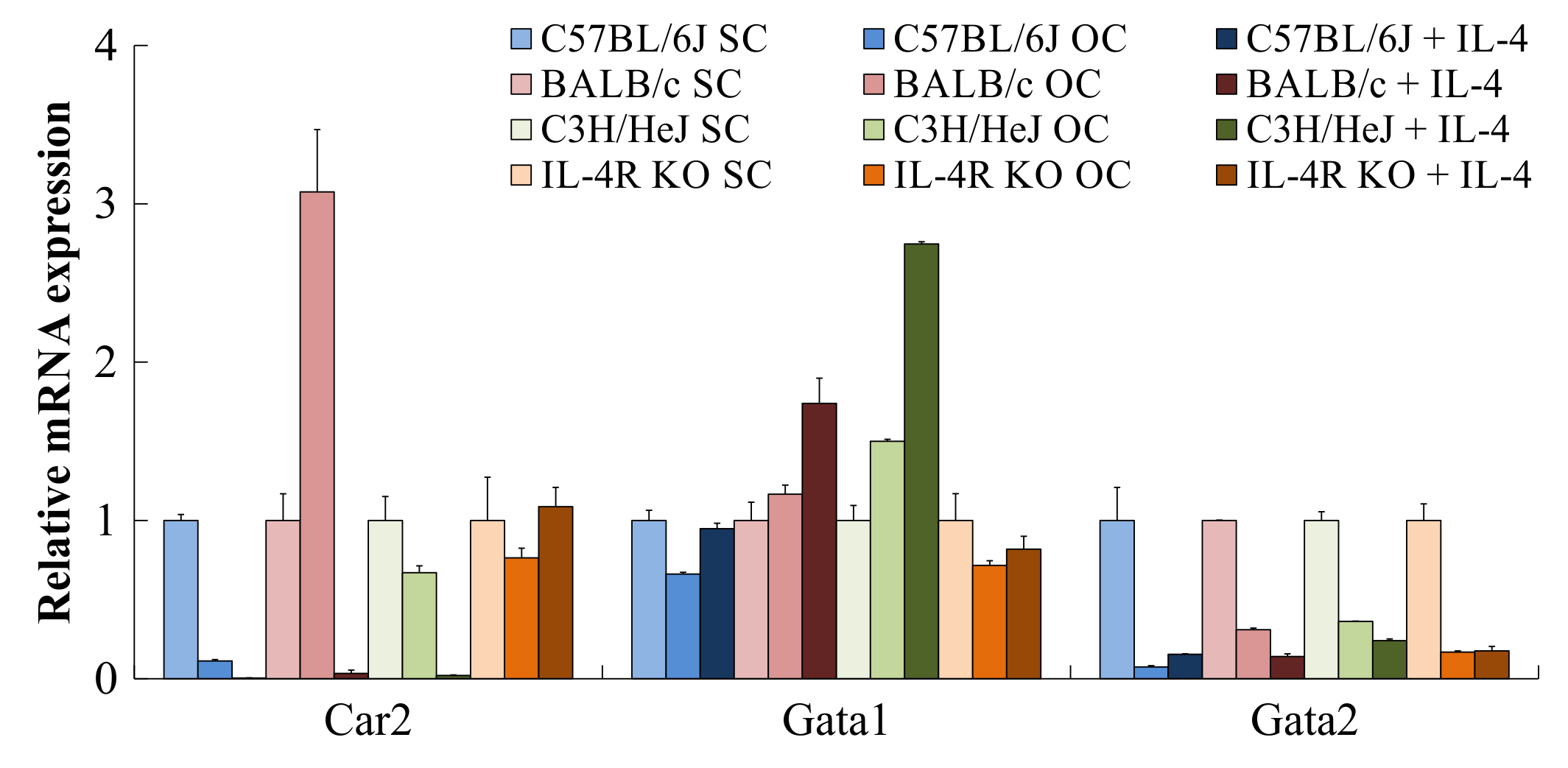

Supplement: S4 Fig — qRT-PCR data of genes Car2, Gata1 and Gata2. RNAs were isolated from ER-Hoxb8 cells of indicated WT or IL-4R KO origin after 5 d under OC differentiation conditions (20,000 cells per cm2). Gene expression of respective SC cultures served as reference control. Data are mean ± SD of technical duplicates. Each sample was pooled together from three 24-wells. Housekeeping gene Hprt was used for normalization of samples. (TIF) [file pone.0142211.s004.tif]
